# Observation of reduced thermal conductivity in a metal-organic framework due to the presence of adsorbates

**DOI:** 10.1038/s41467-020-17822-0

**Published:** 2020-08-11

**Authors:** Hasan Babaei, Mallory E. DeCoster, Minyoung Jeong, Zeinab M. Hassan, Timur Islamoglu, Helmut Baumgart, Alan J. H. McGaughey, Engelbert Redel, Omar K. Farha, Patrick E. Hopkins, Jonathan A. Malen, Christopher E. Wilmer

**Affiliations:** 1 Department of Chemistry, University of California, Berkeley, CA USA; 2 Department of Mechanical and Aerospace Engineering, University of Virginia, Charlottesville, VA USA; 3 Department of Materials Science and Engineering, Carnegie Mellon University, Pittsburgh, PA USA; 4 Institute of Functional Interfaces, Karlsruhe Institute of Technology, Karlsruhe, Germany; 5 Department of Chemistry, Northwestern University, Evanston, IL USA; 6 Department of Electrical and Computer Engineering, Old Dominion University, Norfolk, VA USA; 7 Department of Mechanical Engineering, Carnegie Mellon University, Pittsburgh, PA USA; 8 Department of Materials Science and Engineering, University of Virginia, Charlottesville, VA USA; 9 Department of Physics, University of Virginia, Charlottesville, VA USA; 10 Department of Chemical & Petroleum Engineering, University of Pittsburgh, Pittsburgh, PA USA; 11 Department of Electrical & Computer Engineering, University of Pittsburgh, Pittsburgh, PA USA

**Keywords:** Metal-organic frameworks, Organic-inorganic nanostructures

## Abstract

Whether the presence of adsorbates increases or decreases thermal conductivity in metal-organic frameworks (MOFs) has been an open question. Here we report observations of thermal transport in the metal-organic framework HKUST-1 in the presence of various liquid adsorbates: water, methanol, and ethanol. Experimental thermoreflectance measurements were performed on single crystals and thin films, and theoretical predictions were made using molecular dynamics simulations. We find that the thermal conductivity of HKUST-1 decreases by 40 – 80% depending on the adsorbate, a result that cannot be explained by effective medium approximations. Our findings demonstrate that adsorbates introduce additional phonon scattering in HKUST-1, which particularly shortens the lifetimes of low-frequency phonon modes. As a result, the system thermal conductivity is lowered to a greater extent than the increase expected by the creation of additional heat transfer channels. Finally, we show that thermal diffusivity is even more greatly reduced than thermal conductivity by adsorption.

## Abstract (web summary)

Metal-organic frameworks are attractive adsorbents for gas storage and separations, but appropriate heat dissipation is important for practical application. Here the authors use experiment and theory to show the impact of liquid adsorbates on thermal transport in metal-organic frameworks.

## Introduction

Metal-organic frameworks (MOFs) are a class of porous crystals with extremely high surface areas that have been heralded as promising materials for a wide range of gas storage and separations applications^1–3^. In particular, three major energy applications stand to be improved with appropriately tailored MOFs: (1) hydrogen storage^4–7^, (2) natural gas storage^8,9^, and (3) carbon capture^10–13^. The predominant focus of previous MOF studies has been on the density of the adsorbed gas (usually the higher the better) or the relative difference in adsorbed densities between two or more gas species for separations applications (e.g., high CO_2_ adsorption relative to N_2_ and H_2_O adsorption for carbon capture). However, a critical property for the practical implementation of MOFs for gas adsorption applications has been mostly overlooked: thermal conductivity (*k*).

Because gas adsorption is exothermic, rapid gas loading can result in sharp temperature spikes, which lead to desorption and hence undermine the advantages of using an adsorbent in the first place. A recent study by Wieme et al.^14^ estimated that in the adiabatic limit (i.e., nearly instantaneous gas loading), high pressure CH_4_ or CO_2_ loading can cause temperatures to rise between 100 and 250 K across a range of MOFs. Conversely, rapid gas unloading can result in sharp temperature drops, which prevent the remaining gas from leaving the adsorbent (referred to as “stranded” or “residual” gas). The more slowly the system approaches thermal equilibrium, the longer one must wait to completely fill, or unfill, an adsorbent-containing vessel. Though system dependent, for some applications these fill times can be multiple hours (relative to just a few minutes with no adsorbent). A recent pilot-scale study^15^ considered MOFs and other adsorbents for high-pressure methane storage and found they had to wait two hours for temperatures to equilibrate. This long equilibration time may be a significant concern for any industrial processes where filling or unfilling an adsorbent is a rate-limiting step. It is especially critical for adsorbent-based fuel tanks for methane or hydrogen-powered vehicles, where long fill times may hinder, if not entirely prevent, widespread adoption.

To fulfill their promise as practical industrial gas adsorbents, MOFs need to have both optimal gas adsorption and thermal transport properties. Relative to gas adsorption, however, little is understood about how to tune thermal conductivity in MOFs. Broadly, MOFs are thermal insulators, with thermal conductivities typically <2 W m^−1^ K^−1^ at room-temperature^16–18^. Moreover, it has been an open question as to how adsorbates influence MOF thermal conductivity. This particular question is critical to evaluating MOFs for potential use in gas storage and separations. If adsorbates facilitate thermal transport, then the challenges associated with rapid filling are mitigated, but if they impede thermal transport, the problems are exacerbated.

**Fig. 1:**
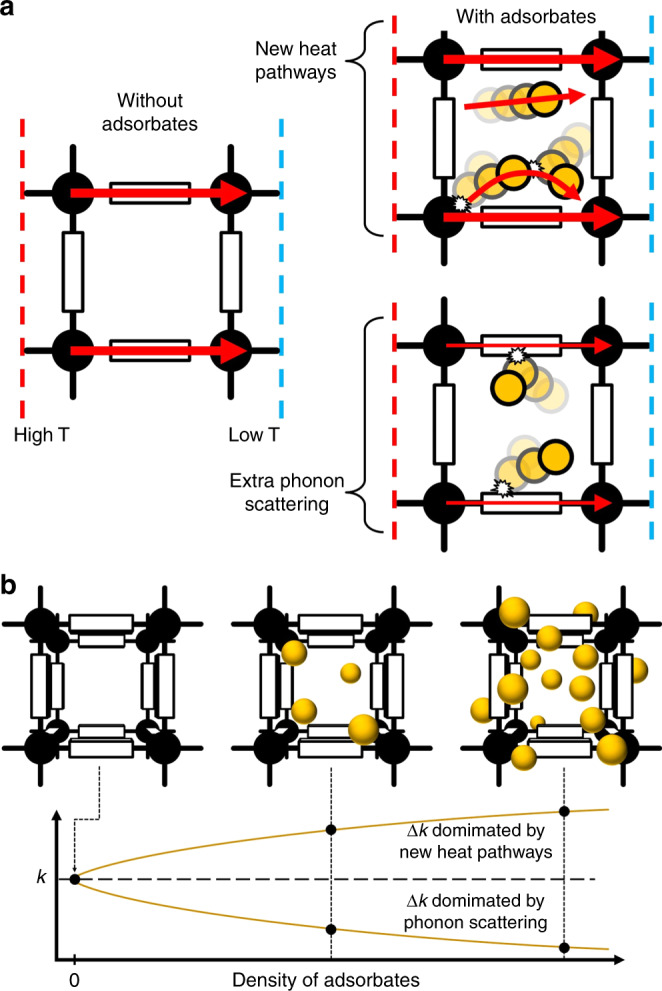
Influence of adsorbates on the thermal conductivity of MOFs. **a** In a vacuum (i.e., without adsorbates), a MOF in a temperature gradient must conduct heat through its framework. However, the presence of adsorbates adds additional pathways for transferring heat and introduces a new source of phonon scattering. **b** As the density of adsorbates in the MOF increases, the system thermal conductivity may increase or decrease depending on whether the effect of new heat pathways or extra phonon scattering is stronger.

Studies of thermal transport in MOFs are limited^16,19–27^. A subset have considered the influence of adsorbates, yet drew conflicting conclusions^21,23,24^. Three of the authors of this article previously suggested, on the basis of molecular simulations, that the presence of an adsorbed gas reduces the thermal conductivity of MOFs due to additional scattering of framework phonons caused by gas-framework collisions^23,28^. Yet another molecular simulation-based study suggested that thermal conductivity increases upon gas adsorption due to additional heat transfer channels^24^. These contrasting descriptions of the adsorbate effect are depicted in Fig. 1 as “new heat pathways” and “extra phonon scattering”.

Experimental evidence regarding the effect of adsorbates on thermal conductivity in MOFs has also been inconclusive^17,21,29^. Schlemminger et al. ^29^ measured the thermal conductivity of MOF-5 powders^30^, compressed into pellets, in the presence of N_2_, He, Ar, and H_2_ gas at various pressures (0.25–15 MPa for H_2_). For higher H_2_ gas loadings, they observed an increase in the system thermal conductivity that was nevertheless lower than the pristine MOF-5 single crystal thermal conductivity. Although thermal transport in powders and pellets, where microcrystalline MOF granules are separated by significant void space, can be representative of behavior in practical adsorbent technologies, these systems provide little insight into the intrinsic nanoscale heat transfer mechanisms taking place within the MOF crystalline phase. Besides powders, thermal transport in the presence of adsorbates has been measured in MOF thin films. Cui et al.^21^ considered N_2_ and C_6_F_14_ adsorption at ambient pressures in thin films of the MOF ZIF-8^31^ and found no significant change in the thermal conductivity. Erickson et al.^17^ measured thermal conductivity in TNCQ-loaded HKUST-1^32^ thin films, but without experimental comparison to the unloaded (i.e., pristine) case.

What has so far been lacking are measurements of thermal conductivity changes within a MOF crystal caused by adsorption. In this article, we report experimental and theoretical investigations of the effect of different adsorbates, including water, methanol, and ethanol, on the thermal conductivity of the MOF HKUST-1 in both single crystal and thin film forms. The experimental measurements were performed independently by labs at Carnegie Mellon University (CMU) and at the University of Virginia (UVA) using two different optical methods: frequency-domain thermoreflectance (FDTR)^33^ and time-domain thermoreflectance (TDTR)^34^, respectively. FDTR measurements were carried out on single crystals synthesized at Northwestern University (NU). TDTR measurements were conducted on thin films prepared at the Karlsruhe Institute of Technology (KIT). The theoretical evaluation was performed using classical molecular dynamics (MD) modeling and the Green–Kubo method for predicting thermal conductivity^23,28^. Both our simulation and experimental results show that the thermal conductivity of HKUST˗1 decreases upon loading with different adsorbate molecules. These results demonstrate that the additional phonon scattering induced by adsorption can dominate the thermal conductivity change by overcoming the creation of additional heat transfer pathways.

## Results

### Methodological overview

Although a driving motivation for this research revolves around the adsorption of gases, we opted to focus the experimental work on liquid adsorbates for two reasons. Firstly, in lieu of performing challenging in situ thermal conductivity measurements at extremely high pressures, pores filled with liquid adsorbates have similar densities to pores with gaseous adsorbates at saturation loading. Secondly, our molecular modeling predictions were insensitive to whether the adsorbate belonged in a liquid or gas phase outside of the MOF (see Supplementary Fig. 1.1). Therefore, if the experiments validated the modeling predictions for liquid adsorbates, the results would provide support for our predictions on gas adsorbates at high pressures. Nevertheless, there may be differences between gas and liquid adsorption in MOFs that are not captured by our molecular models, and assessing the significance of these discrepancies would ultimately require in situ thermal conductivity measurements at high gas pressures.

Equilibrium MD simulations were run at a temperature of 300 K using the large-scale atomic/molecular massively parallel simulator (LAMMPS)^35^ to model HKUST-1 in vacuum and in the presence of liquid water, methanol, and ethanol. The Green–Kubo method^36^ was used to predict the thermal conductivity of each structure (see Supplementary Note 1 and Eq. (S1.1)). We showed in prior work^23,28^ that the trend of adsorption-induced reduction in the thermal conductivity of porous crystals was insensitive to the choice of force field and its parameterization. As such, the objective of our molecular-modeling predictions was to investigate changes in thermal conductivity relative to the pristine case rather than to predict absolute values.

### Experimental setup

**Fig. 2:**
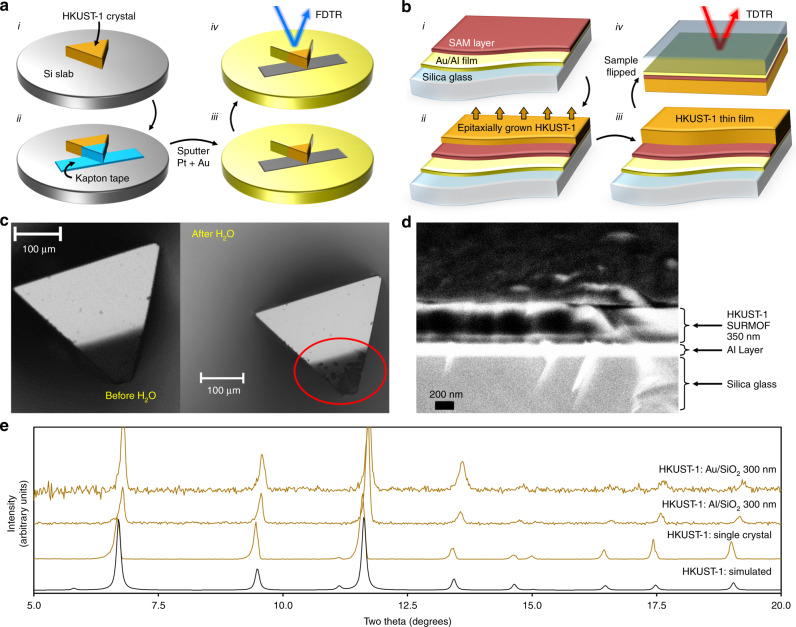
HKUST-1 synthesis, characterization, and thermal conductivity measurements. **a** (i) The HKUST-1 single crystal was glued onto a silicon carrier wafer. (ii) Part of the area was blocked with a strip of Kapton tape. (iii) After sputtering Pt and Au, the tape was removed. (iv) The FDTR lasers were focused on the crystal for thermal conductivity measurements. **b** HKUST-1 SURMOF thin films were prepared by (i) coating an Au or Al surface on a silica substrate with a self-assembled monolayer (SAM) and then (ii and iii) alternately dipping samples in solutions containing either the metal or ligand precursors to grow HKUST-1 epitaxially in layers, followed by (iv) thermal conductivity measurements via TDTR. **c** Optical images of HKUST-1 single crystal before and after soaking in water. **d** HR-SEM image of an HKUST-1 SURMOF film. **e** Simulated and measured PXRD spectra of pristine HKUST-1 bulk crystals and SURMOF films on Au/silica and Al/silica surfaces.

Our parallel experimental measurements on HKUST-1 single crystals and thin films are summarized in Fig. 2. HKUST-1 single crystals, typically ~200 μm in size, were mounted onto a silicon carrier wafer and an Au/Pt transducer layer was sputtered onto them to facilitate thermoreflectance measurements (Fig. 2a). To ensure that liquid adsorbates could enter the crystal freely without being impeded by the Au/Pt transducer layer, ~20% of the area was blocked with a strip of Kapton tape, which was then removed after the transducer layer was sputtered. The partially coated HKUST-1 single crystals were thermally activated, then immersed in methanol, ethanol, or water (see “Methods” section). After removing from the liquid and inspecting optically for signs of degradation (Fig. 2c), each crystal was briefly dried with N_2_ and then measured using FDTR to determine its thermal conductivity. (See Supplementary Note 3 for HKUST-1 crystal synthesis details and Supplementary Note 5 for additional FTDR measurement details.) Separately, polycrystalline HKUST-1 surface-anchored MOF (SURMOF) thin films were self-assembled onto the metal side of Al/silica glass and Au/silica glass substrates using layer-by-layer liquid phase epitaxy^37^ (see Fig. 2b, d and Supplementary Note 2 for SURMOF fabrication details). Powder X-ray diffraction (PXRD) measurements confirmed the same crystal structure on the films as the ~200 μm single crystals (Fig. 2e). The SURMOFs were cut into four sections, three of which were infiltrated with either methanol, ethanol, or water. Only the SURMOF assembled on Au was submerged in water so as to reduce the effects of oxidation likely to occur with the Al in the Al/silica substrates. The thermal conductivities of the pristine and infiltrated HKUST-1 thin films, for a range of film thicknesses, were then measured using TDTR^17,38,39^. See Supplementary Note 4 for TDTR measurement details.

### Thermoreflectance measurements and simulation results

For the pristine HKUST-1 case (i.e., no adsorbates), measured thermal conductivities ranged between 0.44 and 0.73 W m^−1^ K^−1^ across all thin film samples, with the single crystals at the higher end of that range at 0.69 ± 0.05 W m^−1^ K^−1^. These values are in the typical range of other room temperature MOF thermal conductivity measurements, such as IRMOF-1 (0.32 W m^−1^ K^−1^)^16^, ZIF-8 (0.32 W m^−1^ K^−1^)^21^, and perovskite-like MOF-1 (1.3 W m^−1^ K^−1^)^18^. Our single crystal value for HKUST-1 is higher than previously reported by Chae et al. (0.26 W m^−1^ K^−1^)^22^. One possible explanation for this discrepancy is that our HKUST-1 samples were thermally activated for a longer period of time and at higher temperatures (see “Methods” section) than that prior study. If their samples contained residual solvent molecules, a reduced thermal conductivity would be consistent with our observations.

**Fig. 3:**
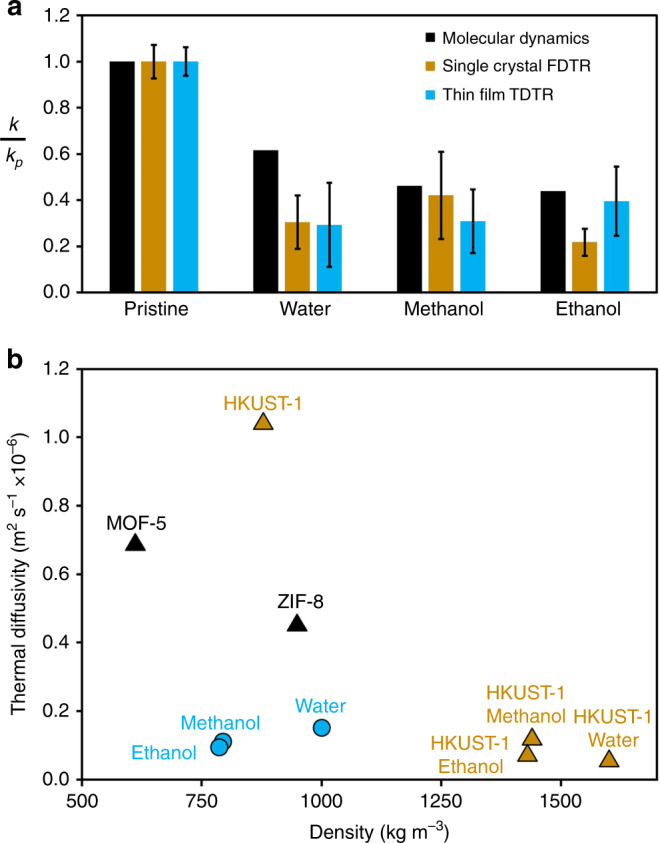
Thermal conductivities and thermal diffusivities of loaded and pristine HKUST-1. **a** Simulation predictions and measurements of thermal conductivity of HKUST-1 loaded with different adsorbates. Depicted is a comparison of normalized thermal conductivities between MD predictions, single crystal FTDR measurements, and TDTR measurements on the SURMOF thin films (300 nm Au for water and 350 nm Al for methanol/ethanol). **b** Thermal diffusivities of HKUST-1 in the pristine state versus the adsorbate-filled state, using FTDR measurements. For comparison, thermal diffusivities of the pure solvents as well as two other MOFs in their pristine state are shown: MOF-5^16^ and ZIF-8^21^. For the purposes of this comparison, HKUST-1, MOF-5, and ZIF-8 were all assumed to have the same heat capacity of 775 J kg^−1^ K^−1^ (see Supplementary Note 5 for how this value was determined).

Both FTDR measurements on HKUST-1 single crystals and TDTR measurements on HKUST-1 thin films followed the same trend as our MD-based predictions: the presence of the selected adsorbates reduced thermal conductivity (Fig. 3a). The reductions ranged from 40% to 80% depending on the adsorbate and methodology. Moreover, the relevant timescale associated with heat dissipation in rapid adsorption applications is inversely proportional to thermal *diffusivity* (*k*/*ρc*, where *ρ* is density and *c* is heat capacity), which is *even more greatly* impacted than thermal conductivity by the presence of these adsorbates (Fig. 3b). In the case of adsorbed water, for example, a threefold thermal conductivity reduction, coupled with a sixfold increase in volumetric heat capacity (*ρc*), leads to an 18-fold reduction in thermal diffusivity. This sensitivity suggests that it may be important to carefully choose MOFs with appropriate thermal properties for rapid gas adsorption applications. As an illustration of the potential impact of this phenomenon, consider the idealized (worst) case where (1) a monolithic single-crystal MOF takes up the entire volume of a natural gas fuel tank (as opposed to the more common form factor of a tightly packed powder), and (2) that gaseous adsorbates at high pressure like methane yield similar thermal conductivity reductions to the liquid adsorbates we measured (as indicated by our simulations, see Supplementary Fig. 1.1b). In this case, if the thermal diffusivity reduction was not taken into account, then adsorption-induced effects would result in filling times more than an order-of-magnitude greater than expected.

These observations support our earlier simulation-based findings that gaseous adsorbates reduce thermal conductivity in porous crystals of varying pore size and shape^23,28^. It appears that the presence of adsorbates in certain MOFs, despite creating more pathways for heat conduction, primarily acts to disrupt thermal transport in the crystalline framework. Our earlier work suggests that the mechanism of this disruption is increased phonon scattering due to adsorbate-framework collisions^23^.

### Origins of adsorbate-induced thermal conductivity reduction

**Fig. 4:**
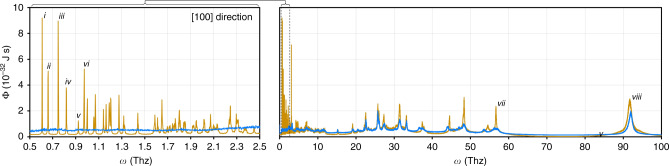
MD-based spectral energy density analysis of pristine and water-loaded HKUST-1. In the pristine case (orange), many sharp peaks are observed at lower frequencies (left) that indicate phonons with long lifetimes. The six lowest frequency phonons (whose peaks are marked i–vi) have lifetimes of 524, 495, 613, 429, 280, and 460 ps, respectively. The lifetimes of the higher frequency phonons (right), by comparison, are much shorter (peaks vii and viii have associated lifetimes of 1.16 and 0.64 ps, respectively), and hence they contribute less to heat transfer within the crystal. When water is adsorbed (*blue*), the low-frequency peaks disappear completely and the higher frequency peaks are dampened. The same pattern is observed for the other two adsorbates: methanol and ethanol (see Supplementary Figs. 1.3 and 1.4).

To elucidate further insights on the adsorbate-induced thermal conductivity reduction in HKUST-1, we performed an MD-based spectral energy density (SED) analysis^40,41^ on the system before and after adsorption (methodology given in the Supplementary Note 1). We found that the effect of the adsorbate in each case (water, methanol, ethanol) was to reduce phonon lifetimes (see Fig. 4 for water and Supplementary Fig. 1.3 for methanol and ethanol). This effect was most pronounced for lower frequency phonons, which had the longest lifetimes prior to adsorption (more than 500 ps) and weaker for higher frequency phonons, whose lifetimes were already very short (1.16 and 0.64 ps for the two highest frequency modes in Fig. 4). It is likely that the long-lifetime, lower-frequency phonons contributed significantly to the thermal conductivity of pristine HKUST-1, and so, when they are strongly scattered by adsorbed species, the system thermal conductivity is diminished. This proposed behavior was directly observed in an MD study of carbon nanotubes that accessed the lifetimes of all phonon modes in empty and water-filled configurations using the SED^41^. Similarly, in the extensive literature on clathrates and skutterudites^42,43^, which are crystals with small but non-interconnected pores/cages that can contain a guest molecule, it has been shown that the presence of a guest molecule reduces thermal conductivity. Though the mechanism is still debated, it is believed that the guest molecule in a clathrate scatters a single phonon mode (or only phonons in a narrow frequency range) due to its constrained motion, which is in contrast to the freely moving adsorbates in our MOF system.

One may reasonably ask if adsorbate-induced thermal conductivity reduction will occur in other MOFs and, if so, under what conditions. If one assumes that the adsorbate phase is a fluid (which may not always be the case, particularly for chemisorption) and we consider only crystalline MOFs (until recent reports of so-called amorphous MOFs^44^, there was no need to make such a distinction), then the adsorbate is necessarily introducing disorder into the system, regardless of the choice of MOF or adsorbate. From the phonon perspective, an adsorbate is a defect that perturbs the potential energy surface of the crystalline framework, introducing additional anharmonicity and thus increasing phonon scattering. It is critical to note that a fluid adsorbate phase can never reduce the phonon scattering rate of a pristine MOF. Hence, the competing factors that influence the MOF thermal conductivity upon fluid adsorption are (i) the degree to which phonon scattering is increased versus (ii) the benefit of gaining additional pathways for heat transfer (see Fig. 1b). For most adsorbates, one can estimate an upper bound for this latter benefit based on its bulk phase thermal conductivity and volume fraction. For the MOF, based on previous theoretical and experimental studies of disordered phases, one can estimate a minimum thermal conductivity by considering the high-scatter limit^45,46^ and previous measurements of ~0.1 W m^−1^ K^−1^. or even more conservatively, zero (assuming the MOF becomes perfectly insulating). From there, one can identify candidate MOF–adsorbate combinations whose thermal conductivity *would increase* with adsorption (i.e., a very insulating MOF with a very conductive adsorbate) and candidates that would behave in the opposite way (i.e., a very conductive MOF with a poorly conducting adsorbate).

In the context of discovering MOFs for rapid adsorption/desorption applications, the adsorbate is typically a given, and the MOF being sought must have, among other properties, high intrinsic thermal conductivity. In such contexts, especially when the adsorbates are gases (which are poor heat conductors), it is likely that MOFs will experience thermal conductivity reductions during adsorption similar to what we observed in this study.

### Thermal conductivity reduction in the adsorbate phase

**Fig. 5:**
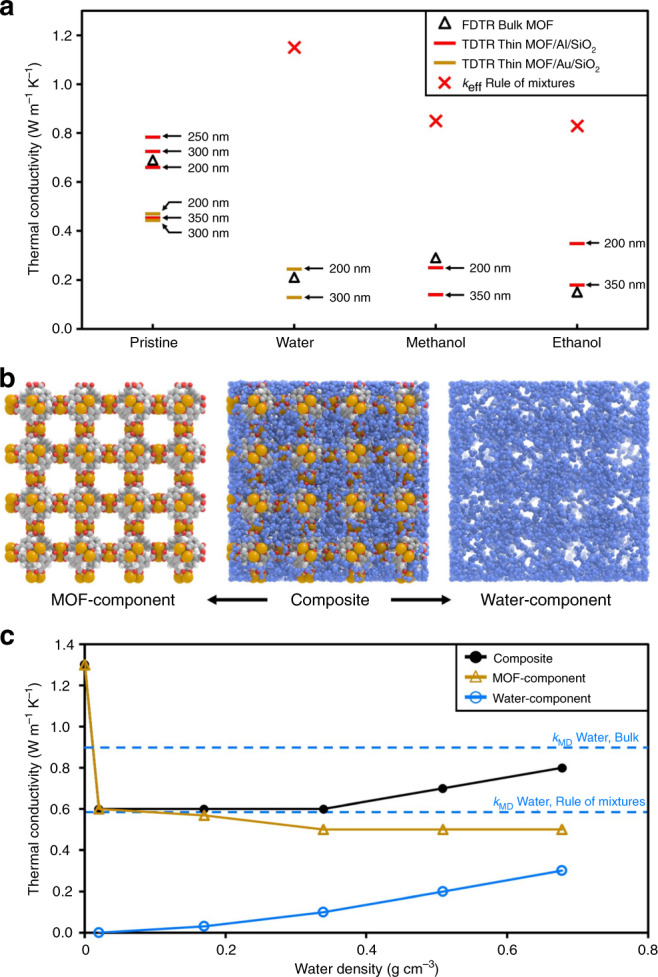
Thin film thermal conductivity measurements and modeling analysis of adsorbed water behavior. **a** Comparison of FTDR and TDTR thermal conductivity measurements to the effective thermal conductivity predicted by the rule-of-mixtures. **b** From the MD simulations, thermal conductivity contributions can be decomposed into water and MOF components. **c** Thermal conductivities of the composite () and individual components (MOF: , water: ) as a function of water density calculated using MD simulations. Note that the thermal conductivity of water in the fully adsorbed MOF is approximately half that expected of bulk water occupying the same contiguous void space.

Surprisingly, our measured thermal conductivity of HKUST-1 with adsorbed water (0.21 ± 0.08 W m^−1^ K^−1^) was not only less than that of pristine HKUST-1 (0.69 ± 0.05 W m^−1^ K^−1^), but was also less than would be expected for bulk liquid water occupying the same contiguous void space (0.6 W m^−1^ K^−1^ × 68% void fraction [*ϕ*] = 0.41 W m^−1^ K^−1^). Thus, not only does the adsorbate reduce the adsorbent MOF’s intrinsic thermal conductivity, but confinement in the MOF pore geometry also reduces the effective thermal conductivity of the adsorbate itself. This finding highlights the inability of effective medium approximations (EMAs) to account for thermal transport in MOF–adsorbate systems. Since MOF–adsorbate composites form bi-continuous structures, any EMA will predict a composite thermal conductivity that is in between that of the MOF (scaled to an equivalent fully dense value: $k_{{\mathrm{fully - dense}}} = k_{{\mathrm{bulk}}} \times \left( {1 - \phi } \right)^{ - 1}$) and the pure phase of the adsorbate^47^. Indeed, even applying a simple rule-of-mixtures (i.e., $k_{{\mathrm{MOF + adsorbate}}} = k_{{\mathrm{MOF,pristine}}} + \phi \times k_{{\mathrm{adsorbate,bulk}}}$) significantly overpredicts the thermal conductivities of HKUST-1 with all tested adsorbates (Fig. 5a).

Further MD simulations suggest that the water inside HKUST-1 has a lower thermal conductivity than bulk liquid water even at the saturation density (Fig. 5b, c). Here we estimated the contribution of water to the thermal conductivity of the MOF–adsorbate composite by considering a system with a rigid MOF (i.e., MOF atoms are frozen and unable to vibrate). In this case, the system thermal conductivity has only contributions from the water phase, since the rigid MOF cannot transfer heat. Subsequently, the intrinsic thermal conductivity of the MOF was obtained by subtracting those contributions of water from the MOF–adsorbate composite thermal conductivity. This procedure indicated that although the thermal conductivity of water inside the MOF increased with adsorption density, even at saturation its thermal conductivity (0.30 W m^−1^ K^−1^) was lower than what would be expected from pure water occupying the same space (the MD-based bulk water thermal conductivity is 0.87 W m^−1^ K^−1^, for which a rule-of-mixtures approach would suggest a value of 0.59 W m^−1^ K^−1^ at a 68% void fraction).

We considered two possible causes of the observed thermal conductivity reduction in the adsorbate phase for water: (1) low connectivity, due to small pore apertures, between the volumes of water contained in neighboring pores of the MOF, and (2) framework-induced disruption of the natural hydrogen-bonding network. Calculations of radial distribution functions (see Supplementary Fig. 1.2) did not show any meaningful change in the water structure in the adsorbed versus pure phases, suggesting that framework-induced disruption of the natural hydrogen-bonding network is an unlikely origin of the reduced thermal conductivity. Hypothetically, if the aperture sizes between the pores of HKUST-1 were reduced to zero, the pockets of water in the pores would be disconnected and unable to transfer heat directly between each other. For the small but non-zero aperture sizes in HKUST-1, the transport of water between pores is restricted in a way that decreases thermal transport in the adsorbed water phase (see Supplementary Note 6 for further details).

Finally, we note that the MD modeling predicts that the thermal conductivity of HKUST-1 drops sharply after only a small amount of water is loaded, with gradual further reductions as the water density increases (see Fig. 5c). This sharp drop suggests that even a small amount of adsorbate loading can, in some cases, contribute significantly to phonon scattering in the framework. At low adsorbate densities, this reduction is likely to depend on the specific nature of the adsorbate–adsorbent interaction (e.g., weakly interacting adsorbates at low densities may not significantly perturb MOF thermal conductivity). This result suggests that optimizing thermal transport may require carefully matching MOF–adsorbate pairs that mitigate such sharp drops in thermal conductivity.

## Discussion

It is important to distinguish these observations of thermal transport within a crystalline MOF from prior, seemingly contradictory, observations of MOF powders^29^, which show an increase in thermal conductivity upon infiltration with gas. Powder thermal resistances are typically dominated by contact between particles, so that the introduction of a gas helps heat to transfer from one particle to another, exemplified by those systems’ pressure-dependent thermal conductivities^48^. Also distinct from MOFs are porous amorphous materials, such as aerogels, which show an increased thermal conductivity with gas pressure^49^. Aerogels have significantly higher porosities compared to MOFs ($\phi \, > \, 99\%$). As such, their pristine thermal conductivities are very low (≈0.01 W m^−1^ K^−1^). Furthermore, unlike MOFs, their large pores are comparable to, or larger, than the bulk gas molecule mean free paths^50^. As such, the molecules of a gas that have infiltrated an aerogel scatter with the solid infrequently and can have a significant thermal conductivity of their own.

As a broad material class, the generality of adsorbate-induced thermal conductivity reduction in MOFs is still to be determined; it is possible that a MOF with a sufficiently low thermal conductivity in combination with highly conductive adsorbate may behave in an opposite way. However, our experimental observations of HKUST-1 with liquid adsorbates are the first direct evidence of this phenomenon and our molecular simulations suggest that certain gaseous adsorbates at high pressure would have a similar impact on thermal conductivity.

There is no doubt that MOFs are remarkable materials that have attracted excitement and attention from a rapidly growing community of scientists. Much of that excitement is based on the promise of MOFs for revolutionizing gas storage and separations applications, particularly for certain key energy and environmental challenges (e.g., hydrogen and natural gas storage, carbon capture). As these materials advance towards implementation outside of research labs, increased scrutiny will be placed on previously neglected properties, such as thermal transport. Our observations indicate potential challenges ahead for certain MOF–adsorbate combinations. The already typically low thermal conductivities of MOFs may be pushed even lower by adsorbates, which exacerbates the challenge of dissipating heat generated during adsorption. Compounding this issue, we found that thermal diffusivity is reduced to an even greater extent than thermal conductivity. If MOF-based adsorbent systems are designed without considering how the adsorbate influences thermal properties, loading and unloading times could be significantly underestimated. This could lead to negative effects for otherwise promising applications, such as vehicular methane and hydrogen gas storage, where fuel tanks must be refilled quickly, as well as for a wide range of industrial separations where long loading times could become rate limiting.

## Methods

### MD modeling

Equilibrium MD simulations were run at a temperature of 300 K using the LAMMPS^35^ to model HKUST-1 in vacuum and in the presence of liquid water, methanol, and ethanol, as well as high-pressure hydrogen and methane gases (see Supplementary Fig. 1.1). The Green–Kubo method^36^ was used to predict the thermal conductivity of each structure (see Supplementary Note 1 and Eq. (S1.1)). To obtain the adsorbate densities, grand canonical Monte Carlo (GCMC) simulations were performed using the RASPA software package^51^ at a pressure of 1 bar for liquid adsorbates and 35 and 100 bar for methane and hydrogen, respectively. For both MD and GCMC, force fields were used without any parameter fitting specific to this work; all parameters were developed by others (see more details in the Supplementary Note 1). We showed in prior work^23,28^ that the trend of adsorption-induced reduction in the thermal conductivity of porous crystals was insensitive to the choice of force field and its parameterization. As such, our objective was to apply molecular modeling to investigate changes in thermal conductivity relative to the pristine case, rather than to predict absolute values.

### Single crystal HKUST-1 measurements using FDTR

HKUST-1 single crystals were synthesized, activated under vacuum at 150 °C, and characterized at NU (see Supplementary Note 3 for synthesis details). Crystals from the same batch were also sent in non-activated form to CMU and activated using the same procedure. The samples, typically ~200 μm in size, were mounted onto a silicon carrier wafer and a Au/Pt transducer layer was sputtered over them to facilitate thermoreflectance measurements (Fig. 3a). To ensure that liquid adsorbates could enter the crystal freely without being impeded by the Au/Pt transducer layer, ~20% of the area was blocked with a strip of Kapton tape, which was then removed after the transducer layer was sputtered. To measure how the thermal conductivity changed with liquid adsorption, the partially coated HKUST-1 single crystals were immersed in methanol or ethanol for 1 h, or in water for 20 min, immediately following the thermal activation process. These immersion times were found to yield full (i.e., saturation) adsorption in a prior study^52^. After removing each crystal sample from the liquid, inspecting optically for signs of degradation (Fig. 3c), and briefly drying their surface with N_2_, FDTR measurements were conducted at room temperature to determine their thermal conductivities. The FDTR lasers were scanned across the crystal until a spot was identified that was sufficiently smooth and flat for the required optical reflectance. (See Supplementary Note 5 for more FTDR measurement details.)

### Thin film HKUST-1 measurements using TDTR

Polycrystalline HKUST-1 SURMOF thin films were self-assembled onto the metal side of Al/silica glass and Au/silica glass substrates using layer-by layer liquid phase epitaxy^37^ (see Fig. 3b and Supplementary Note 2 for SURMOF fabrication details). PXRD measurements confirmed the same crystal structure on the films as the ~200 μm single crystals synthesized at NU (Fig. 3e). At UVa, the pristine HKUST-1 SURMOF samples were activated^52,53^ by vacuum thermal annealing at a pressure of 4.5 × 10^-6^ mbar and a temperature of 150 °C for 12 h. The activated SURMOFs were cut into four sections. Each section was separately infiltrated with methanol, ethanol, and water. As with the single-crystal samples used for FTDR measurements, the SURMOF films were fully submerged for an hour in each of the polar organic solvents and for 20 min in water. Only the SURMOF assembled on Au was submerged in deionized water, so as to reduce the effects of oxidation likely to occur with the Al in the Al/SiO_2_ substrates. The thermal conductivities of the HKUST-1 thin films in activated pristine form and loaded with different solvents for a range of film thicknesses were then measured using TDTR^17,38,39^ (Fig. 3g). (See Supplementary Note 4 for more TDTR measurement details.)

## Supplementary information

Supplementary Information

## Data Availability

The data that support the findings of this study are available from the corresponding author upon request.
