# Supplementary material for: Observation of reduced thermal conductivity in a metal-organic framework due to the presence of adsorbates
Source: Nat Commun. 2020 Aug 11;11:4010. doi: 10.1038/s41467-020-17822-0 (PMC7421542; doi:10.1038/s41467-020-17822-0)
Supplement: Supplementary file 1 — Supplementary Information [file 41467_2020_17822_MOESM1_ESM.pdf]

Supplementary Information

Observation of reduced thermal conductivity in a metal-organic framework due to the presence of adsorbates

Hasan Babaei,[a] Mallory E. DeCoster,[b] Minyoung Jeong,[c] Zeinab M. Hassan,[d] Timur Islamoglu,[e] Helmut Baumgart,[f] Alan J. H. McGaughey,[c,g] Redel Engelbert,[d] Omar K. Farha,[e] Patrick E. Hopkins,[b,h,i] Jonathan A. Malen,[c,g] and Christopher E. Wilmer[j,k*]

[a] Department of Chemistry, University of California, Berkeley, CA, USA
[b] Department of Mechanical and Aerospace Engineering, University of Virginia, Charlottesville, VA, USA
[c] Department of Materials Science and Engineering, Carnegie Mellon University, Pittsburgh, PA, USA
[d] Institute of Functional Interfaces, Karlsruhe Institute of Technology, Karlsruhe, Germany
[e] Department of Chemistry, Northwestern University, Evanston, IL, USA
[f] Department of Electrical and Computer Engineering, Old Dominion University, Newport News, VA, USA
[g] Department of Mechanical Engineering, Carnegie Mellon University, Pittsburgh, PA, USA
[h] Department of Materials Science and Engineering, University of Virginia, Charlottesville, VA, USA
[i] Department of Physics, University of Virginia, Charlottesville, VA, USA
[j] Department of Chemical & Petroleum Engineering, University of Pittsburgh, Pittsburgh, PA, USA
[k] Department of Electrical & Computer Engineering, University of Pittsburgh, Pittsburgh, PA, USA

Contents

Supplementary Note 1. Molecular dynamics (MD) simulations **pg 2**

Supplementary Note 2. Fabrication of HKUST-1 thin films (SURMOFs) **pg 9**

Supplementary Note 3. Fabrication of HKUST-1 single crystals **pg 14**

Supplementary Note 4. Time-domain thermoreflectance (TDTR) measurements **pg 16**

Supplementary Note 5. Frequency-domain thermoreflectance (FDTR) measurements **pg 23**

Supplementary Note 6. Sintered sphere model for thermal conductivity **pg 30**

* Corresponding author. E-mail address: wilmer@pitt.edu

Supplementary Note 1. Molecular dynamics (MD) simulations

Force field

HKUST-1 has a simple cubic structure with 624 atoms in the unit cell and an experimentally-measured lattice constant at room temperature of 2.63 nm.[1] HKUST-1 contains large pores of diameter 10 Å connected by channels of diameter 3.5 Å to smaller tetrahedral pores of diameter 5 Å. For the interactions between atoms in HKUST-1, we used the force field developed by Zhao et al.[2] The simulation cell size was set based on the experimental lattice constant. This value was also used in the development of the force field for HKUST-1 by Zhao et al.[2] The initial atomic configurations for the MD simulations involving adsorbates were taken from snapshots of equilibrated Grand canonical Monte Carlo (GCMC) calculations[3] at different pressures (details are provided below). The adsorbate-filled MOF structures were not relaxed. The average pressure in all simulations were between 4000 and 6500 bar, including the pristine case.

Different adsorbates were simulated including water, methane, hydrogen, methanol, and ethanol. Methane was modeled as a point particle with the TraPPE force field.[4] Methanol and ethanol were also modeled using the TraPPE force field for alcohols.[5] The potential for methanol contains three sites (CH_2, O, and H), with bond stretching and bending potentials between sites. The potential for ethanol contains an additional site (CH_3) and a dihedral term. Water was modeled using a modified version[6] of the SPC/E potential[7] that considers fully-flexible intramolecular interactions. For hydrogen, a three-site model including charges on all sites and an LJ interaction on the center of mass developed by Darkrim and Levesque was used.[8] An LJ cutoff distance of 12 Å was used for all simulations. Lorentz–Berthelot mixing rules[9] were used to model the van der Waals interactions between the adsorbate and the framework atoms. The electrostatic interactions were handled using the Particle–Particle–Particle–Mesh (PPPM) solver with a relative error in the forces of 10^{-5}. The MD simulations were performed using the Large-scale Atomic/Molecular Massively Parallel Simulator (LAMMPS)[10] software with a time step of 0.5 fs and with periodic boundary conditions applied in all directions.

Green-Kubo calculation of thermal conductivity

The Green-Kubo formula for thermal conductivity is[11]

$$k_{ii} = \frac{V}{k_B T^2} \int_0^\infty < J_i(t) J_i(0) > dt, \quad i = x, y \text{ or } z. \tag{S1.1}$$

Here, the i-th diagonal element of the thermal conductivity tensor (k_{ii}) at temperature T is calculated by integrating the heat current autocorrelation function (HCACF) over time. The HCACF is extracted from

equilibrium molecular dynamics (MD) simulations. In Eq. (S1.1), k_B is the Boltzmann constant, V is the volume of the simulation box that contains the system of particles and $\boldsymbol{J}$(t) is the microscopic heat current. To obtain thermal conductivity values, we averaged over the *x*-, *y*-, and *z*-directions.

The microscopic heat current is calculated from[11]

$$\mathbf{J}(t) = \frac{1}{V}\left[\sum_{j=1}^{N}\mathbf{v}_j E_j - \sum_{\alpha=1}^{2} h_\alpha \sum_{j=1}^{N_\alpha}\mathbf{v}_{\alpha j}\right] + \frac{1}{V}\left[\frac{1}{2}\sum_{i=1}^{N}\sum_{j=1, j\neq i}^{N}\mathbf{r}_{ij}\left(\mathbf{v}_j.\mathbf{F}_{ij}\right)\right], \quad \text{(S1.2)}$$

where $\mathbf{v}_j$ and E_j are the velocity vector and instantaneous energy of particle *j*. The quantities $\mathbf{r}_{ij}$ and $\mathbf{F}_{ij}$ are the displacement vector and interacting force between particles *i* and *j*. The parameter *N* is the total number of particles and N_α is the number of particles for species α. h_α denotes the average partial enthalpy of species α and is given by

$$h_\alpha = \frac{\sum_{i=1}^{N_\alpha}\left[K_i + U_i + \frac{1}{3}\left(m_i v_i^2 + \frac{1}{2}\sum_{j=1}^{N}\mathbf{r}_{ij}.\mathbf{F}_{ij}\right)\right]}{N_\alpha}, \quad \text{(S1.3)}$$

where K_i and U_i are the time-averaged kinetic and potential energies of particles of species α.

For determining thermal conductivity, a system size of 2×2×2 unit cells was used. The systems were initially equilibrated under *NVT* conditions at a temperature of 300 K for 300,000 time steps and further equilibrated for 300,000 time steps under *NVE* conditions. Finally, *NVE* simulations were run for an additional 1,000,000 time steps where the heat current was calculated every 10 time steps. For all cases, we performed this procedure for eight simulations starting from random velocity distributions. The HCACFs were calculated using a correlation time of 50 ps and then averaged and integrated for the thermal conductivity prediction. Thermal conductivity values were obtained from the plateau region of the HCACF integral (see Eq. S1.1) that corresponded to 20 ps (the 20-40 ps time window) for the pristine MOF and less than 40 ps (the last 40 ps) for the loaded MOFs.

Grand canonical Monte Carlo simulations

GCMC simulations were performed using the RASPA simulation software[12] to determine the amount of adsorbed methane, hydrogen, water, methanol, and ethanol in HKUST-1. All GCMC simulations were performed at a temperature of 300 K.

All GCMC simulations of adsorption included an M-cycle equilibration period followed by an M-cycle production run, where M was 5000. A cycle consists of n Monte Carlo steps; where n is equal to the average number of molecules. All simulations included random insertion, deletion, translation, and rotation moves of molecules with equal probabilities. Atoms belonging to the crystal structure were held fixed at their crystallographic positions.

For the methane loading, pressures of 1 and 35 bar were considered. For water, pressures of 0.1 and 1 bar were considered and the rest of the considered water densities were fractions (1/4, 1/2, and 3/4) of that at the highest water density (at 1 bar). For methanol and ethanol loading, a pressure of 1 bar was used, while for hydrogen a pressure of 100 bar was used.

Heat capacity calculation

The heat capacity of HKUST-1 required in the TDTR and FDTR thermal conductivity measurements was calculated using harmonic lattice dynamics calculations as implemented in the GULP software package[13]. In this method, the heat capacity is calculated using $C = \sum_{\omega} \frac{\hbar\omega}{V} g(\omega) \frac{\partial f_{BE}}{\partial T}$ in which g is the phonon density of states, ω is the phonon frequency, and f_{BE} is the Bose-Einstein distribution. The calculated heat capacity at a temperature of 300 K is 780 J/kg-K. We note that the heat capacity calculated using lattice dynamics in the classical limit is consistent that from our MD simulations based on $C = (\langle E^2 \rangle - \langle E \rangle^2)/Vk_B T^2$.

For the loaded cases, effective medium theory was used to calculate the required densities and heat capacities under the assumption of fully loaded pores at a void fraction of 0.7. The volumetric heat capacity and density of a loaded MOF are calculated as $C = C_{MOF} + \varphi C_{adsorbate}$ and $\rho = \rho_{MOF} + \varphi \rho_{adsorbate}$ where C_{MOF} and ρ_{MOF} are the volumetric heat capacity and density of the MOF and $C_{adsorbate}$ and $\rho_{adsorbate}$ are the volumetric heat capacity and density of adsorbate. φ is the void fraction. The specific heat capacity is calculated by dividing C by ρ.

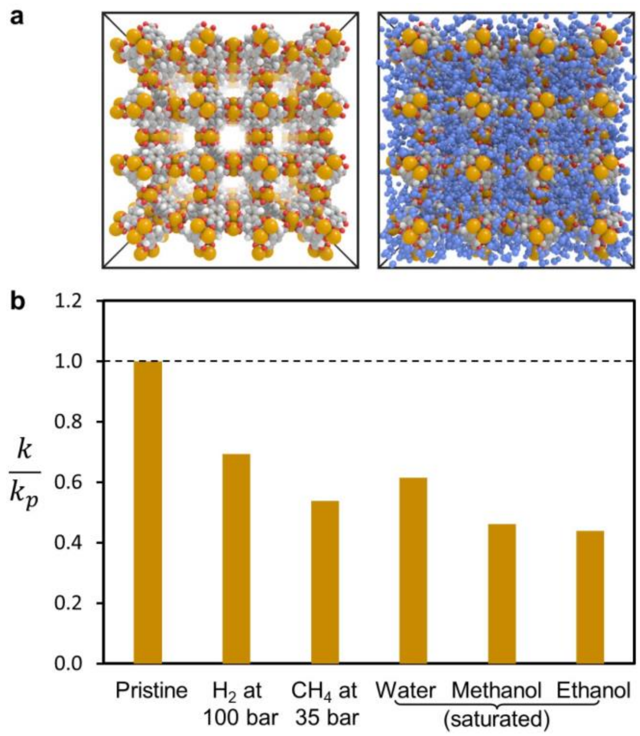

Supplementary Figure 1.1. Predictions of reduced thermal conductivity in HKUST-1. **a,** MD simulations were performed on HKUST-1 with and without various adsorbates. Depicted are snapshots of an HKUST-1 simulation box (*left*) without adsorbed water and (*right*) water at saturation loading (liquid density). **b,** In all cases, whether the pores were saturated with gas or liquid adsorbates, we observed a decrease in thermal conductivity. Here k_p refers to the thermal conductivity of pristine HKUST-1.

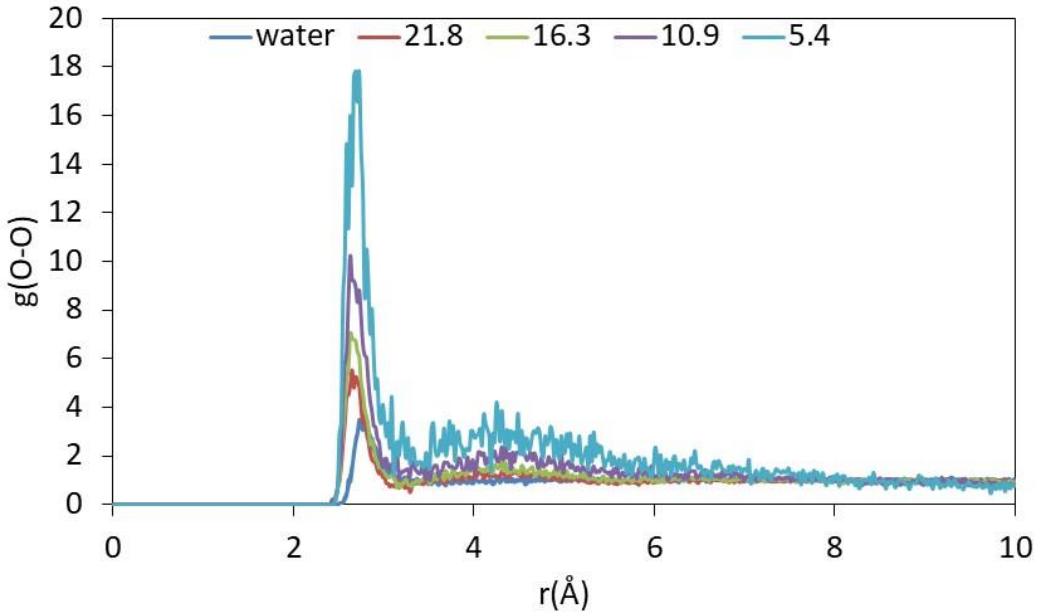

Supplementary Figure 1.2. Radial distribution function (RDF) of adsorbed water in HKUST-1 at various densities. The distribution labelled "water" corresponds to bulk water, and the other distributions are labelled by the number of water molecules per unit cell of HKUST-1 (with 21.8 corresponding to the saturation loading case).

Spectral energy density calculation of phonon frequencies and lifetimes

We applied the spectral energy density (SED) method[14] to calculate phonon lifetimes. In this method, the SED is calculated from

$$\Phi(K,\omega) = \frac{1}{4\tau_0\pi}\sum_{\alpha,\beta}\frac{m_b}{N}\left|\int_0^{\tau_0}\sum_l \dot{u}_\alpha\binom{l}{b},t)\, exp(\,i\boldsymbol{K}\bullet\boldsymbol{r}_l - i\omega t)dt\right|^2 \quad \text{(S1.4)}$$

where $\boldsymbol{K}$ and ω are the wave vector and frequency. τ_0 is the integration time, which should be larger than the largest mode lifetime, N is the number of unit cells in the supercell, m_b is the mass of atom b in the unit cell, $\dot{u}_\alpha\left(\binom{l}{b},t\right)$ is the α -th component of velocity of atom b in unit cell l, and r_l is the position vector for unit cell l.

The calculated SED for each $\boldsymbol{K}$ contains a number of peaks that is equal to the number of phonon branches in the dispersion curve (3 times the number of atoms in the unit cell). The lifetime of each phonon mode is equal to the inverse of the full-width at half-maximum (FWHM) of a Lorentzian function fit to its peak. In all SED calculations, we saved the velocities of all atoms every 5 fs in production EMD simulations run for 1,000,000 time steps (time step=1 fs). SED curves for the [100], [110], [111], and [000] directions, for all 3 adsorbates + the pristine case, are shown in Supplementary Figure 1.3. We fitted

Lorentzian peaks to the 6 lowest and 2 highest modes for the [100] direction in the pristine system to get phonon lifetimes (see fits in Supplementary Figure 1.4.).

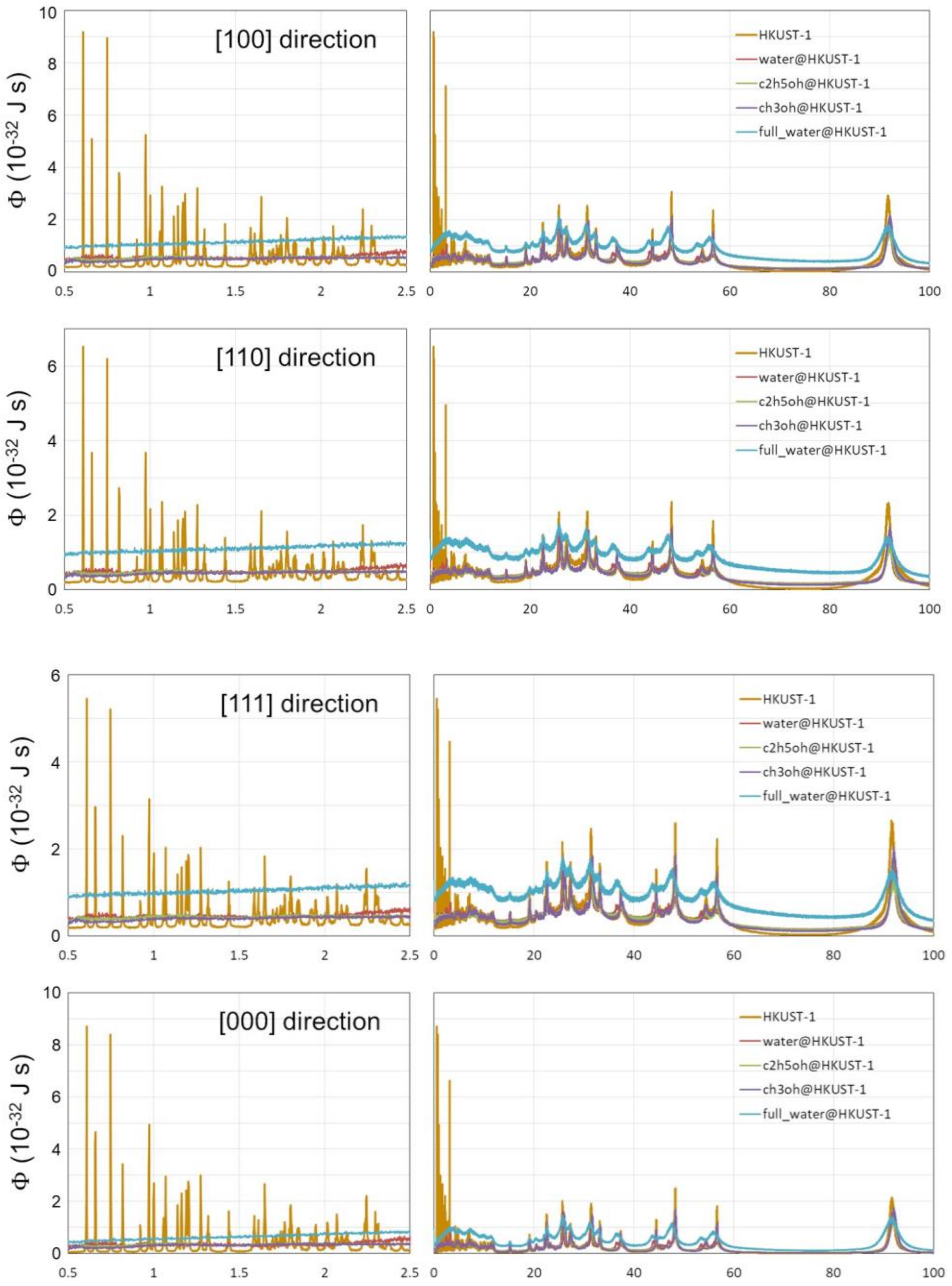

Supplementary Figure 1.3. SED for the [100], [110], [111], and [000] directions, for all 3 adsorbates and the pristine case (no adsorbates). Two different loadings for water are shown, saturation density (*full water*) corresponding to 21.8 water molecules per nm^3, and a low density (*water*) of 0.66 water molecules per nm^3.

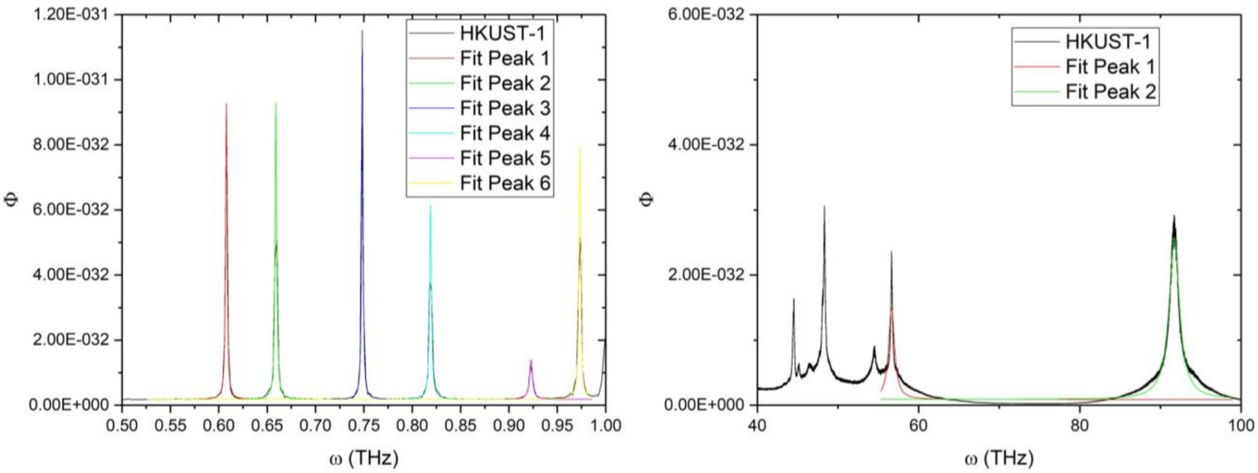

Supplementary Figure 1.4. Fitted Lorentzian peaks to the 6 lowest and 2 highest modes for the [100] direction in the pristine system.

Supplementary Note 2. Fabrication of HKUST-1 thin films (SURMOFs)

$Cu_3(BTC)_2$ SURMOFs were grown on a modified Au/Al substrate on SiO_2 using the LBL-method[15]. Before deposition, surface modification and cleaning of the Au/Al substrates was carried out using O_2 plasma cleaning[16]. The HKUST-1 SURMOFs used in this work were fabricated using a spray system[17] for $Cu_3(BTC)_2$ as described in detail in an earlier publication[18]. The spray times were 15 s for the copper acetate solution (1 mM) and 25 s for the BTC solution (0.2 mM). Each spray step was followed by a rinsing step (5 s) with pure ethanol to remove residual reactants. A total of 90 cycles (≈ 200 nm), 150 cycles (≈ 300 nm) and 190 cycles (≈ 350 nm) hand-spray growth cycles were used for all $Cu_3(BTC)_2$ SURMOFs samples investigated in this work. Before further processing, all SURMOFs samples were activated by ultrasonication in dichloromethane solution for 2 min to remove residual solvents from the SURMOFs pores and characterized by X-ray diffraction (XRD). The XRD, IRRAS and UV-Vis results obtained for the $Cu_3(BTC)_2$ SURMOFs are characteristic and comparable to already published work in the field[19,20].

SURMOF characterization - X-ray Diffraction (XRD)

Each sample was characterized by using a Bruker D8 Advance equipped with a Si-strip detector (PSD Lynxeye©; position sensitive detector) with Cu $K_{\alpha 1,2}$ radiation (λ = 0.15418 nm) in θ–θ geometry, variable slit on primary circle. Scans were run over various ranges with step width of 0.024° 2θ and 84 seconds, for higher order peaks up to 336 seconds per step. The 2θ angle scanning range to observe corresponding peak to deposited film is picked up from 5° to 60°.

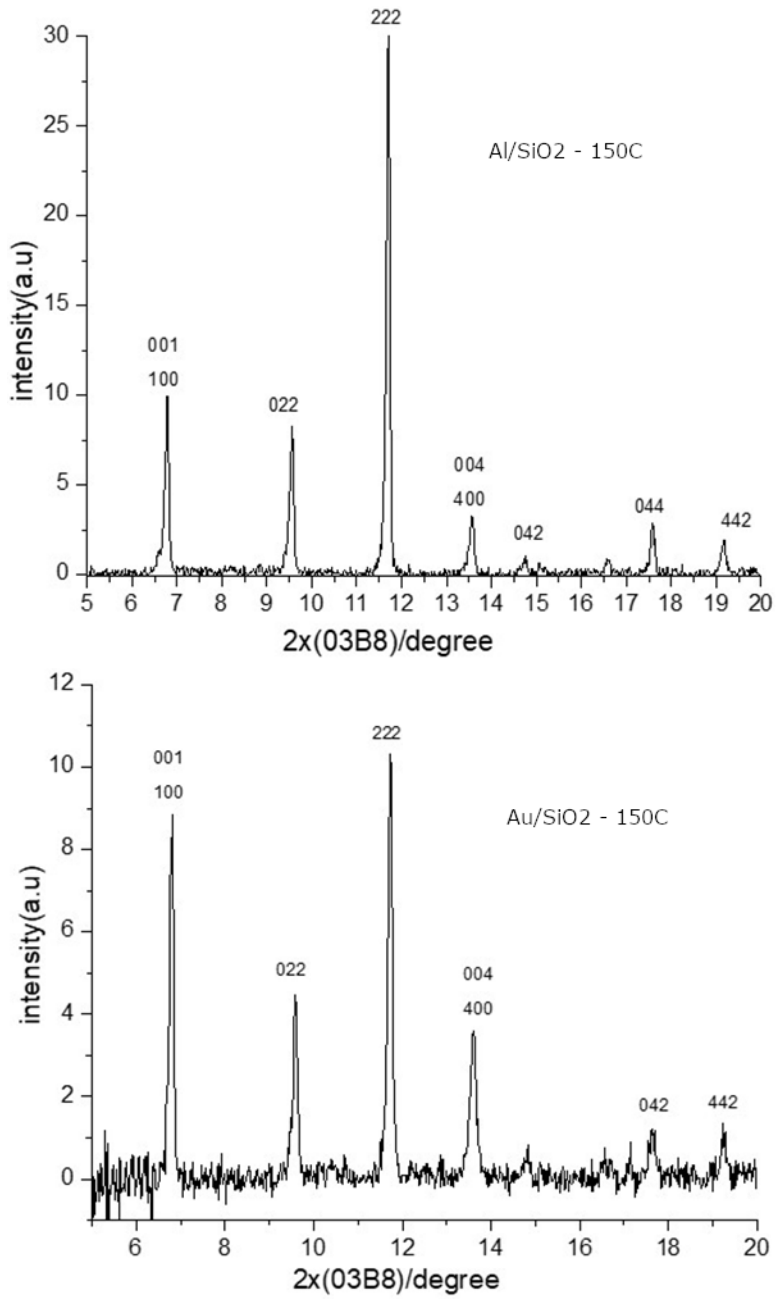

Supplementary Figure 2.1 PXRD of HKUST-1 SURMOF thin films 150C (≈ 300 nm) on Al/Si as well as on Au/quartz substrates.

SURMOF characterization - Infrared reflection absorption (IRRA) spectroscopy

IRRA spectra were measured using the infrared spectrometer (Bruker VERTEX 80) purged with dried air. The spectra were recorded in grazing incidence reflection mode at a fixed angle of incidence of 80° relative to the surface normal using mercury cadmium telluride (MCT) detector. Predeuteratedhexadecanethiol SAM on Au/Ti/Quartz substrates were used for reference measurements.

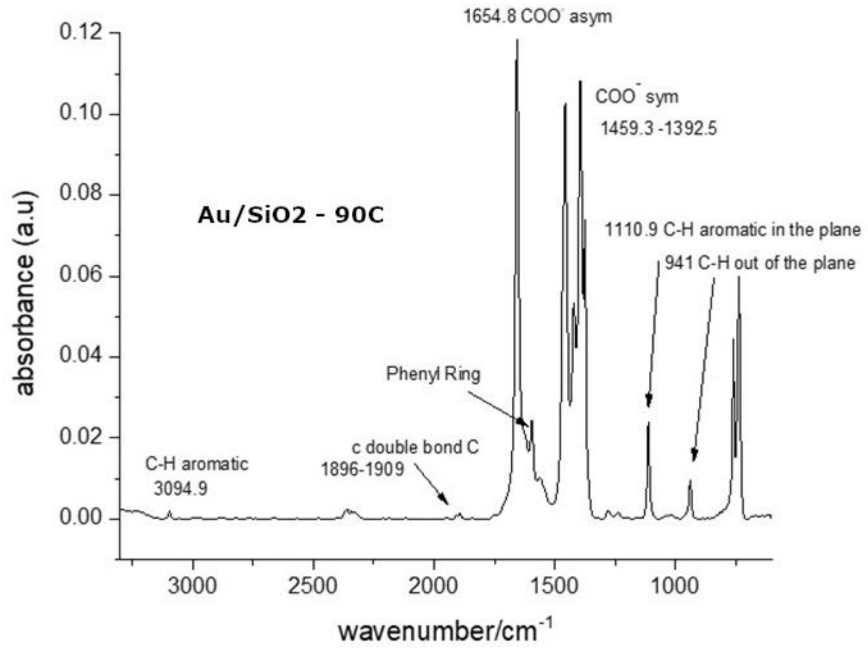

Supplementary Figure 2.2 IRRAS of SURMOFs thin films 90C (≈ 200 nm) on Al/Si as well as on Au/quartz substrates.

SURMOF characterization - Scanning electron microscope (SEM)

HR-SEM cross-sectional measurements have been performed on a Zeiss HR-SEM (Gemini Class) at 3-5 kV to check the continuity, compactness, and homogeneity of the different prepared (loaded and unloaded) HKUST-1 thin films (see Fig. 2 in manuscript and Supplementary Fig. 2.3 below).

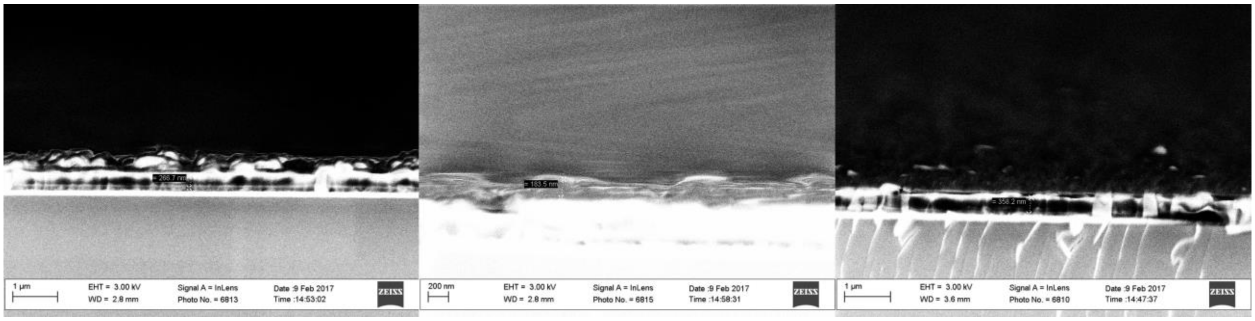

Supplementary Figure 2.3 HR-SEM images of prepared HKUST-1 SURMOFs of varying thicknesses.

SURMOF characterization - Quartz microbalance (QCM)

The mass changes as well as the uptake of guest molecules in the HKUST-1 SURMOF have been recorded by a QCM Model QE401-F1206 from Q-Sense (LOT Distributor). Data evaluation have been performed with Q-Software from Q-Sense. The HKUST-1 SURMOFs were grown on 16-mercaptohexadecaonic acid (MHDA) functionalized QCM sensors using the quasi-LPE method. For functionalization, the sensors were rinsed by pure ethanol then dried in a dry N_2 stream and kept in a 20µM ethanolic MHDA solution for three days. Subsequently, the sensor was removed and rinsed with pure ethanol. The QCM cell was equipped with a sensor and the frequency was monitored by the system. To build HKUST-1 SURMOF 1 mM of ethanolic solution of Cu $(CH_3COO)_2.H_2O$ and BTC 0.2 mM ethanolic solution of linker (benzene-1,3,5-tricarboxylic acid). The metal solution was run for 8 min then rinsed by pure ethanol for 4 min followed by linker for 10 min then rinsed by pure ethanol for 4 min, this is one cycle. The cycle was repeated for 200 times. Finally, the formed HKUST-1 was rinsed with pure ethanol for 14 hours. So, the thickness was build up in around 230 nm. The crystalline structure of the HKUST-1 on QCM sensor was confirmed by X-ray diffraction. The diffraction reflexes at 6.7° (200), 13.4° (400), demonstrate the crystallinity of the HKUST-1 SURMOF as well as the oriented growth in [200] orientation. The mass change of the sample on the QCM sensor was determined using the the Sauerbrey equation ($\Delta m = -A \cdot \Delta f$; A is a device specific parameter, in this case 17.7 ng cm^{-2} Hz^{-1}). The sample thickness was determined from the change of the resonance frequency Δf during the synthesis. A SURMOF mass of 31µg/cm^{-2} was determined, which corresponds to a thickness of about 230 nm considering the density of ethanol filled HKUST-1 is about 1.53g cm^{-3}.

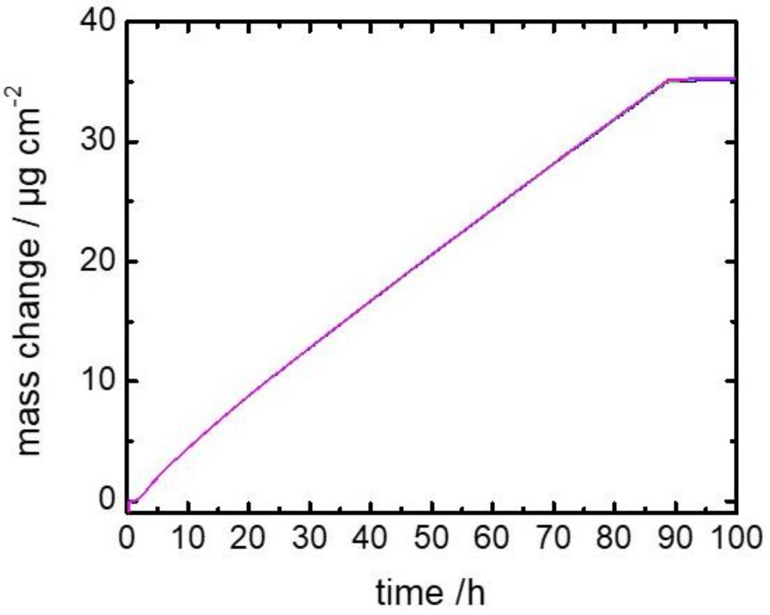

Supplementary Figure 2.4 Growing of HKUST-1 SURMOF determined by QCM

The uptake of water was performed using a setup described in prior work[21]. The setup may be shortly explained, a gas system using argon as a carrier gas is connected to the QCM cell to allow flow through the cell. For uptake experiments the flow is diverted to pass through a washing bottle filled with water. The uptake of H_2O in the gas phase is performed by connecting a gas flow system to the QCM cell. A flow rate of 99 mL min^{-1} Ar flow was used as the carrier gas in the experiment. Before the adsorption of probe molecule, a stable baseline was achieved under Ar flow at 60°C. The argon flow is firstly enriched with the vapor of the H_2O by passing through the reservoir of the guest molecules. H_2O possesses a saturation vapor pressure of about 3.1 kPa at room temperature[22]. The enriched gas flow is then passed through the QCM cell, where the guest molecules diffuse into the SURMOF permitting the uptake to be studied by the mass change of the SURMOF on the QCM sensor. It should be noted that the concentration of argon in MOFs of type HKUST-1 at 60 ˚C and a pressure of about 1 bar is very small. Therefore, the argon molecules in the pores as well as the diffusion of argon during the uptake of the guest molecules can be neglected and the activated MOF pores can be considered as empty.

The flow rates of the gases through the QCM cell need to be set large enough to ensure that the uptake by the SURMOF in gas phase is controlled by diffusion in the nanopores and not by convection through the QCM cell. In this case, 99 mL min^{-1} is set for the gas flow. Switching from the pure gas flow to the enriched gas flow, results in the uptake of the guest molecules into HKUST-1 SURMOF. The mass changes are recorded by the QCM. The uptake of H_2O was repeated several times to avoid the possible deviation. All experiments were performed at a temperature of 60 °C. Before each experiment, the sample was activated in a flow of pure argon at 60 °C for several hours, to guarantee that no molecules, such as ethanol, or water were adsorbed in the pores and to guarantee reproducible results. Calculation yielded a filling of around 120 water molecules per unit cell. Then after calculation, we found that the big pore of HKUST-1 was uptake around 20 molecules, and the whole unit cell absorbed around 120 molecules of H_2O.

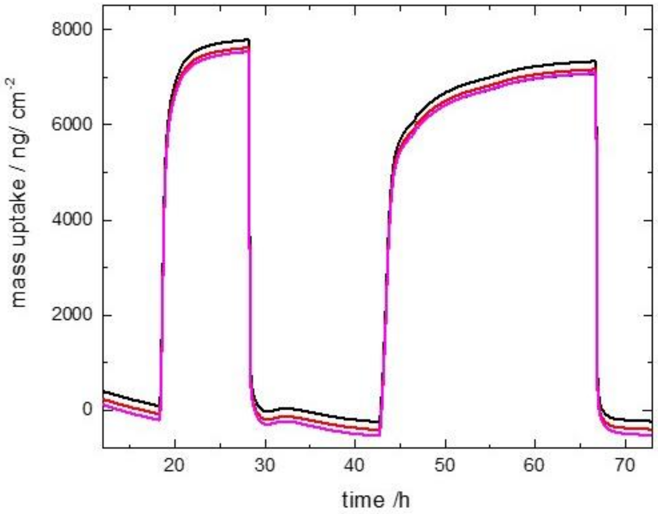

Supplementary Figure 2.5 Uptake and release of H_2O over time in HKUST-1 SURMOF monitored by QCM

Supplementary Note 3. Fabrication of HKUST-1 single crystals

Synthesis of HKUST-1

In an 8 dram 1,3,5-benzenetricarboxylate (500 mg, 2.38 mmol) and oxalic acid dihydrate (60 mg, 0.48 mmol) were dissolved in 10 mL of EtOH and 1 mL DMF mixture. In a separate vial $Cu(NO_3)_2{\cdot}3H_2O$ (1.65 g, 6.8 mmol) was dissolved in 9 mL of H_2O and was added to linker solution. The resulting milky suspension was sonicated and placed in 80 °C pre-heated oven for 48 h. The insoluble white precipitate can be easily removed using density separation by adding fresh ethanol and pipetting out the white suspension above the crystals. This can be repeated until the white suspension is completely removed. The resulting blue HKUST-1 crystals were further washed with ethanol and dried under vacuum at 150 °C prior to N_2 isotherm measurements.

Characterization details

Nitrogen isotherm measurements were carried out on a Micromeritics Tristar II 3020 at 77 K. Samples were activated at 100 °C for 1 h and 150 °C for 16 h under vacuum on Micromeritics Smart VacPrep instrument and outgas rate below 0.05 mmHg/min was achieved. Pore size distribution was calculated using DFT model provided by MicroActive software (Micromeritics). The calculated BET surface area for HKUST-1 was 1805 m2/g and the total pore volume was 0.6 cc/g.

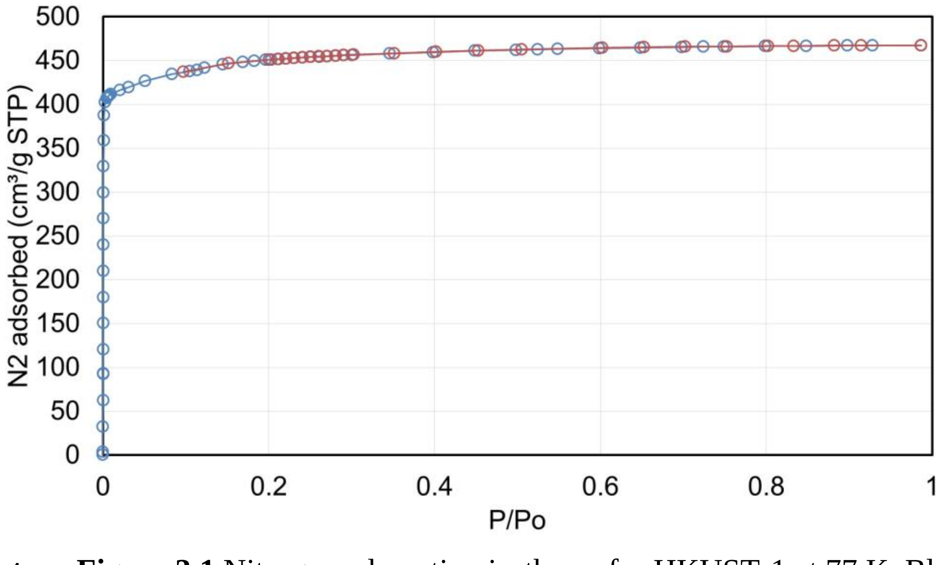

Supplementary Figure 3.1 Nitrogen adsorption isotherm for HKUST-1 at 77 K. Blue circles = adsorption, orange circles = desorption.

Powder X-ray diffraction (PXRD) of HKUST-1 was measured at room temperature on a STOE-STADIMP powder diffractometer equipped with an asymmetric curved Germanium monochromator

(CuKα1 radiation, λ = 1.54056 Å) and one-dimensional silicon strip detector (MYTHEN2 1K from DECTRIS). The line focused Cu X-ray tube was operated at 40 kV and 40 mA. The activated powder was sandwiched between two Kapton foils and measured in transmission geometry in a rotating holder. Intensity data from 2 to 55 degrees two theta were collected over a period of 15 min. The instrument was calibrated against a NIST Silicon standard (640d) prior to the measurement.

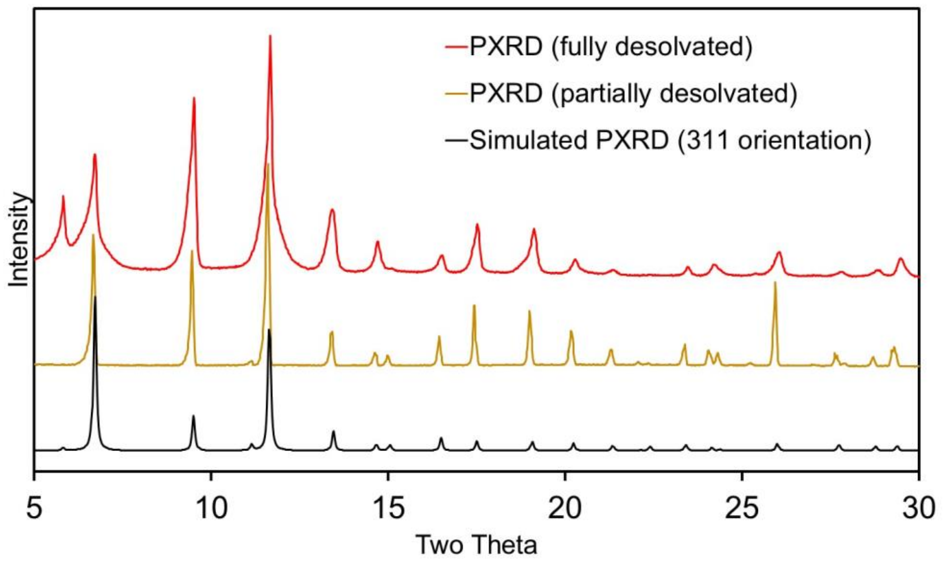

Supplementary Figure 3.2 Powder X-ray diffraction (PXRD) of large HKUST-1 single crystals. We collected two PXRD patterns from the same batch of crystals, where in one case they were fully desolvated (*red*) and in the other partially desolvated (*orange*) for which the peaks are much sharper. The simulated PXRD shown here (*black*) was made with preferred orientation on the (311) plane (as opposed to the simulated PXRD shown in the manuscript that used no preferred orientation setting).

Supplementary Note 4. Time-domain thermoreflectance (TDTR) measurements

Experimental Analysis

This section offers an in depth description of the experimental and analysis procedures used to obtain the thermal conductivities of thin film pristine and infiltrated SURMOFs with time domain thermoreflectance (TDTR)[23–26]. The Al/SiO_2 and Au/SiO_2 substrates fabricated for this study consisted of 80 nm of Al or 75 nm of Au electron-beam evaporated onto bulk amorphous SiO_2 substrates at a base pressure of 2×10^{-6} Torr. The metal layer served to transduce the optical energy from the TDTR laser pulses into temperature excursions within the sample; the frequency-dependent temporal decay of which was then measured. The Au/SiO_2 substrates were fabricated in addition to the Al/SiO_2 substrates to rule out the effects of additional reactions that the aluminum coated samples may have experienced when exposed to water, along with any transducer or metal/SURMOF interface effects on the measured SURMOF thermal conductivity.

TDTR is a non-contact, optical thermometry technique that utilizes a pump-probe experimental configuration. A Ti:Sapphire femtosecond Spectra Physics Tsunami oscillator emits sub-picosecond pulses at a repetition rate of 80 MHz with a wavelength centered at ~800 nm (bandwidth of 10.5 nm). The output of the Ti:Sapphire laser is split into a pump and probe path. The pump path is electro-optically modulated with a square wave. The pump beam passes through the transparent SiO_2 substrate and heats the aluminum or gold layer, which creates a modulated heating event at the sample surface. The heat transfers to both the SiO_2 and the SURMOF film. The reflectivity of the aluminum (or gold) changes linearly with surface temperature and is monitored temporally by the probe beam. The probe is delayed in time by a translational mechanical delay stage up to 5.5 ns, and its reflected intensity from the sample is measured by a photodetector. Since this change in reflectivity of the probe pulses due to pump heating is very small (on the order of 10^{-4} – 10^{-6}), a lock-in amplifier is used to demodulate the signal and provides amplitude and phase data in the form of a thermal decay curve which is analyzed with an appropriate thermal model to derive the thermal properties of interest[17,18,21]. In this sample configuration, bi-directional heat conduction must be accounted for in the analysis[20]. The heat conduction model used to fit the data requires knowledge of the heat capacity, thermal conductivity, and thickness of all of the material making up the sample. Literature values are assumed for the heat capacity of the aluminum, gold, and SiO_2[28]. We experimentally measure the thermal conductivity of the SiO_2 ($\kappa = 1.35\ Wm^{-1}K^{-1}$) and the thermal boundary conductance between the transducer and SiO_2 layer ($G_{Au/SiO2} = 4.7 \times 10^7$ $Wm^{-2}K^{-1}$ and we are insensitive to the thermal boundary conductance across the Al/SiO_2 interface) with TDTR in a standard geometry (incident on the air/metal side of the metal/glass sample) before the MOF are deposited onto the metal coated side of the metal/SiO_2 substrates. The model is very sensitive to the film thickness

of the metal film transducers, which were each measured via profilometry and/or picosecond acoustics on the exact substrates for each MOF (Supplementary Table 4.1 below). The thermal conductivities of the metal films (κ_{Al}=120 W m^{-1}K^{-1}, κ_{Au} = 312 W m^{-1}K^{-1}) were experimentally obtained using four point probe electrical resistivity and TDTR measurements. Lastly, the thicknesses of the MOFs were confirmed through SEM after they were deposited onto the SiO_2 substrates.

Supplementary Table 4.1: The thickness of the metal transducer layer measured for each sample before the MOF was deposited.

Sample	MOF Thickness (nm)	Tranducer Thickness (nm)
SiO_2/Au/MOF	200	75
SiO_2/Au/MOF	300	78
SiO_2/Al/MOF	200	82
SiO_2/Al/MOF	250	83
SiO_2/Al/MOF	300	82
SiO_2/Al/MOF	350	82

The layer thicknesses of the aluminum and gold are confirmed by picosecond acoustics[29] and profilometry, respectively, and the thickness of the SURMOFs are confirmed through SEM. We take advantage of the TDTR experimental configuration to measure both the thermal conductivity and heat capacity of the thinnest pristine SURMOF films. This is accomplished by performing TDTR measurements at a pump modulation frequency of 8.8 MHz, where the signal is sensitive to both the heat capacity and thermal conductivity, and analyzing both the in-phase and the ratio of the in-phase to out-of-phase components of the probe thermoreflectivity generated by the lock-in amplifier[30,31]. Supplementary Fig. 4.1 a) and c) show plots of the TDTR signal sensitivity to the thermal conductivity and heat capacity of a 200 nm activated pristine HKUST-1 at a pump modulation frequency of 8.8 MHz. Specifically, Supplementary Fig. 4.1 a) shows the TDTR sensitivity of the in-phase component of the TDTR signal (V_{in}) and b) shows the sensitivity of the ratio of the in-phase to the out-of-phase components (V_{in}/V_{out}) of the TDTR signal to the thermal conductivity and heat capacity to the HKUST-1 SURMOF on both Al/SiO_2 and Au/SiO_2 substrates[32]. The sensitivity calculations are a measure of how strongly the thermal model depends on the parameter (i.e. thermal conductivity of the MOF) being measured compared to the other parameters in the system (i.e. heat capacity of the transducer, TBC between the transducer and SiO_2 substrate, etc.). Supplementary Fig. 4.1 b) and d) display the normalized residuals[29] (Z) resulting from the model's fit relative to the obtained thermal decay curve (r) for a single TDTR scan from SiO_2/Au/HKUST-1 given perturbations in alternative parameters of the thermal conductivity (κ) and heat capacity (C_v).

$$Z = \left(\frac{\sum_{x_0}[r(x_o;\kappa_{exact}),C_{v,exact} - r(x_o;\kappa_{perterbed},C_{v,perterbed})]^2}{\sum_{x_0} r(x_o;\kappa_{exact}))^2}\right)^{1/2} \quad \text{(S4.1)}$$

The contour plots indicate the range of values that would receive a good fit given the value used for the heat capacity or thermal conductivity. The thick black contour line represents a 3% residual error, where all values inside that contour line represent the acceptable range of thermal conductivities based on a single heat capacity value resulting from a good fit of the thermal model to the data. We see that the in-phase component of the signal is insensitive to the heat capacity. Therefore we perform an iteration procedure where we first fit the in-phase component of the TDTR signal for the thermal conductivity (where the input value for the heat capacity will not affect the result for the thermal conductivity) and then we use that value as an input to our model and fit the ratio for both thermal conductivity and heat capacity. We iterate between these two methods until the value for thermal conductivity converges.

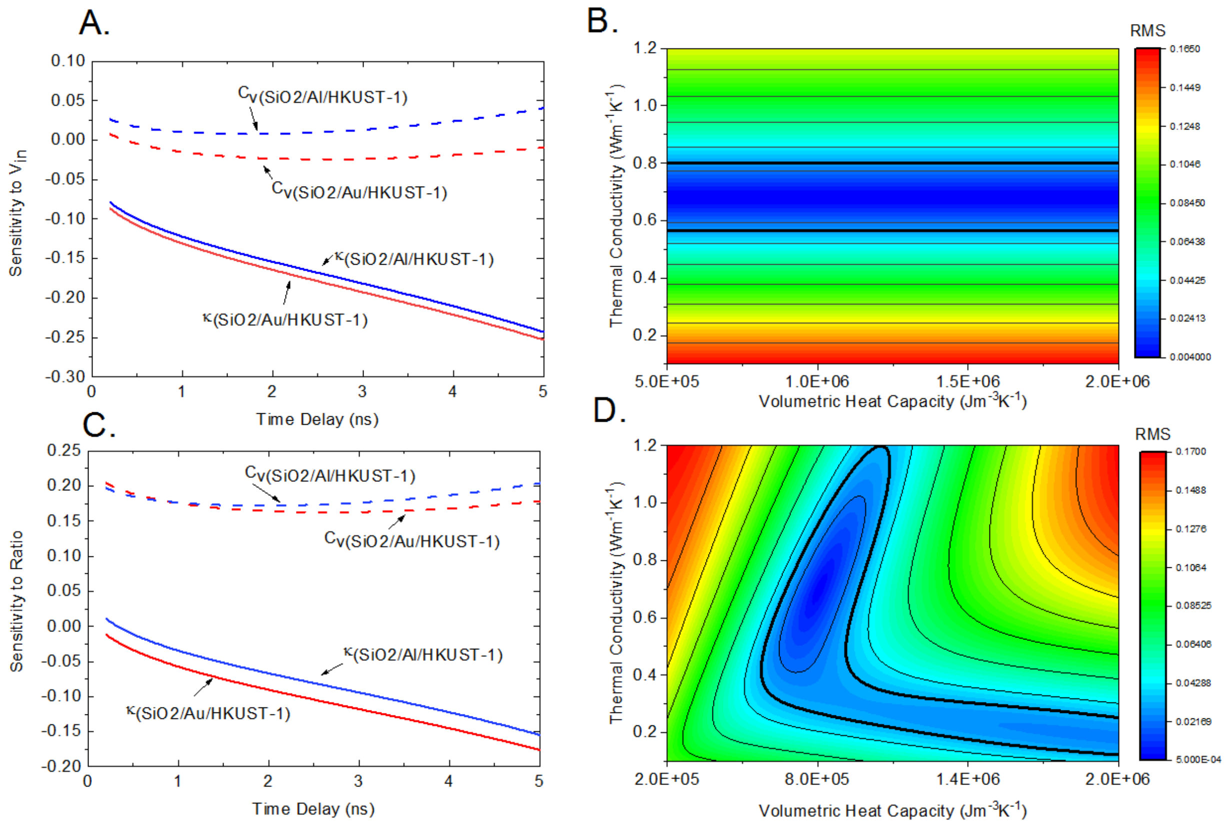

Supplementary Figure 4.1 Sensitivity plots for the thermal conductivity and heat capacity of the 200 nm pristine SURMOF grown on both Al and Au to the a) V_{in} (in-phase) component of the TDTR signal, and to the c) ratio ($-V_{in}/V_{out}$) of the TDTR signal at 8.8 MHz modulation frequency. The contour plots show the residual (the model fit compared to the obtained thermal decay curve) for a single TDTR scan for a 200 nm SiO_2/Au/HKUST-1 fit to b) the V_{in} (in phase) signal and d) the ratio. The plots show that the V_{in} signal is insensitive to heat capacity. The thick black contour line represents an error of 3% which indicates a good fit of the thermal heat transport model to the TDTR data.

We measure a pristine activated HKUST-1 volumetric heat capacity of 8.1×10^5 J m^{-3} K^{-1} (see Supplementary Figure S4.2). The TDTR signal's sensitivity to both thermal conductivity and heat capacity at a single modulation frequency is limited to the thinnest pristine HKUST-1 MOF films (200 and 250

nm). For our SURMOF sample thicknesses considered in this work, we do not expect the heat capacity to depend on thickness at room temperature, and therefore we use the volumetric heat capacity measured for the 200 and 250 nm pristine HKUST-1 samples as an input to the thermal model for the thicker SURMOF samples. Our thermal analysis can also be very sensitive to the thermal boundary conductance between the metal and glass layer. Thus, we measure the thermal boundary conductance (G) across the Au/SiO_2 interface before SURMOF deposition as $G_{Au/SiO2}$= 4.7x10^7 +/- 9.2x10^6 W m^{-2} K^{-1}; we are insensitive to the thermal boundary conductance across the Al/SiO_2 interface due to its relatively low resistance relative to the thermal resistance of the SiO_2 substrate. The measured values for the heat capacity of the SURMOF films are within 18% of the calculated values (see SI page 4). Slight variations between our measured values for the SURMOF heat capacity and the computationally-predicted values are reasonable since it is expected that our SURMOF thin films will not be in a perfectly ideal state which is captured in the simulations. Supplementary Fig. 4.2 compares the measured and calculated heat capacity values. The uncertainty in the heat capacity and thermal conductivity measurements arrives from ±3 nm uncertainty in the Al and Au film thicknesses, as well as uncertainties in slight differences in the SURMOF sample, as scans were rastered across the surface. In total, the uncertainties in the thermal properties that were assumed (either though literature values or measurements) for the metal transducer and SiO_2 substrate increase the uncertainty in the measured thermal conductivity and heat capacities of our MOF films. We use an iterative method of fitting for the thermal conductivity to the in-phase (V_{in}) signal, where we are relatively insensitive to the heat capacity of the MOF (for example, changing the thermal conductivity of the MOF by 20% results in a change of the heat capacity of 1%), and then fitting for the ratio ($-V_{in}/V_{out}$) for the heat capacity of the MOF. Further, to accurately determine the uncertainty in our multivariable TDTR measurements, we considered the mean square deviation of our bi-directional, four-layer thermal model to the experimental data using a contour analysis uncertainty calculation for data fit to both the in-phase (V_{in}) and ratio ($-V_{in}/V_{out}$). The contour plot (Supplementary Fig. 4.1) displays the thermal model's mean square deviation relative to experimentally measured thermal decay curves (equation S4.1). A large RMS value corresponds to a poor fit of the model to the data. We constrain our goodness of fit criteria to represent an error of <3%, indicating a good fit of the model to the data. The total uncertainty in our measurements account for the scan to scan variability on different spots of the sample, the uncertainty in the goodness of fit of our thermal model to our data, and the uncertainty of the metal transducer thickness. The metal transducer thickness is the most sensitive parameter and propagates the most uncertainty into our thermal measurements. The measured thermal decay curves and the best fit heat conduction model for the 200 nm pristine SURMOF sample are shown in Supplementary Fig. 4.3.

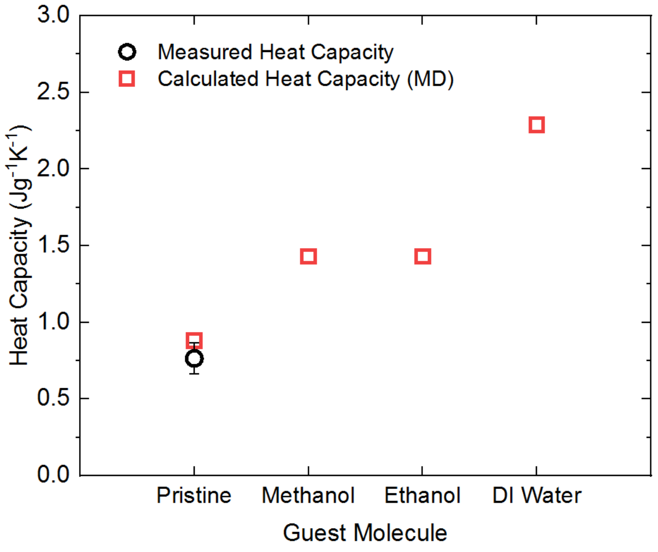

Supplementary Figure 4.2 Molecular dynamics calculated heat capacity vs. TDTR measured gravimetric heat capacity of SURMOF thin films. Only the pristine film was measured because the TDTR experiment did not have sensitivity to the heat capacity of the guest@HKUST-1 samples.

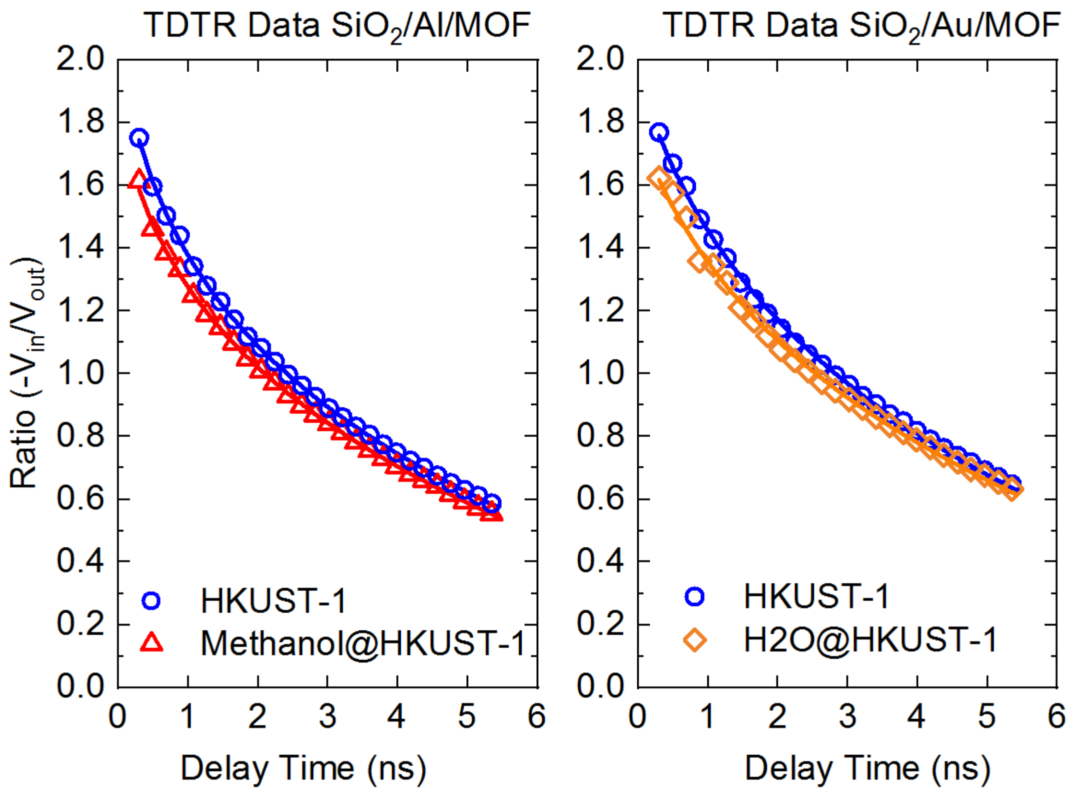

Supplementary Figure 4.3 Representative data for a TDTR scan on a 200 nm thick SURMOF deposited on an Al/SiO_2 substrate (left) and Au/ SiO_2 (right), measured at a pump modulation frequency of 8.8 MHz, and analyzed with a four layer model for pristine HKUST-1, methanol@HKUST-1, and H2O@HKUST-1. The ethanol@HKUST-1 data is excluded for clarity.

The thermal conductivity vs. film thickness data are plotted in Supplementary Fig. 4.5. The thermal conductivity is independent of film thickness, where size effects do not seem to play a role in reducing the thin film thermal conductivity compared to the bulk FDTR sample results. In addition, we note that that the SURMOFs grown on the SiO_2/Au substrates show slightly reduced thermal conductivities compared to the SURMOFs grown on SiO_2/Al substrates. This could be due to differences in the polycrystalline nature of the SURMOFs that is dictated by the Al or Au layer. As shown in Supplementary Fig. 2.1, XRD data indicate that both SURMOF systems are polycrystalline, however higher intensity peaks within the SiO_2/Al/HKUST-1 SURMOFs are indicative of larger grain sizes leading to fewer boundaries within the SURMOF material. We perform a Scherrer analysis on the XRD data to further understand the differences in polycrystallinity and strain within the samples.

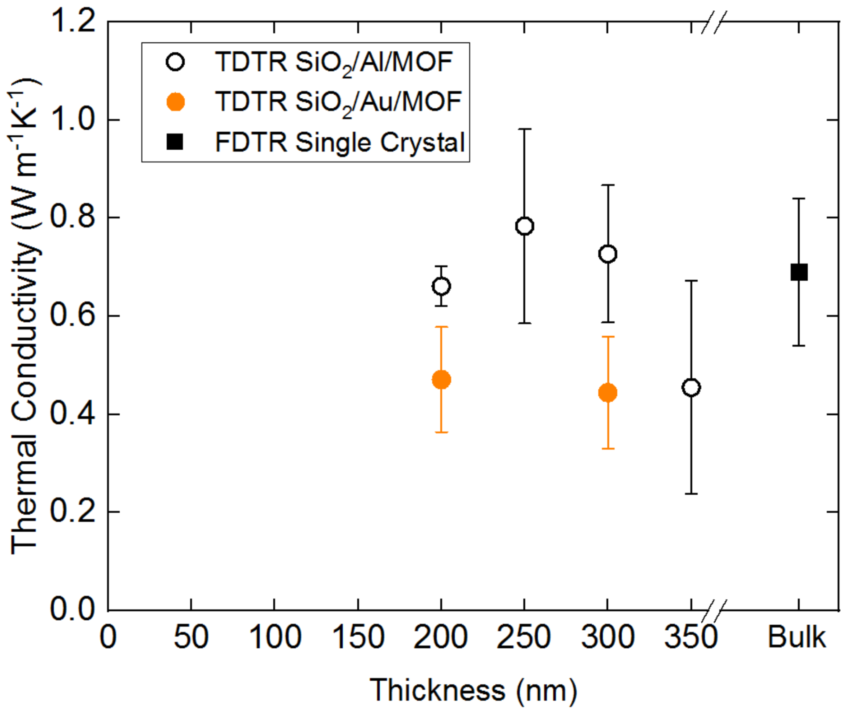

Supplementary Figure 4.5 Thermal conductivity vs. SURMOF film thickness compared to the bulk FDTR data.

Scherrer Equation for analyzing XRD data

In order to better characterize the structural differences between the HKUST-1 MOFs grown on Au and Al substrates we determine the size of the crystallite domains and the strain within the samples using Williamson-Hall plots and the Scherrer equation[26]. This method works by taking advantage of the fact that the broadening of XRD peaks is due to three features: broadening that is intrinsic to the XRD instrument, contributions from strain, and the size of the ordered crystalline domains. The broadening due to the instrument is determined by analyzing a gold calibration sample. The broadening due to the effects of crystallite size and strain are represented by the following equations respectively:

$$\beta_L = \frac{K\lambda}{L\cos(\theta)}, \qquad \text{(S4.2)}$$

$$\beta_e = C\varepsilon \tan(\theta) \quad \text{(S4.3)}$$

Where K is the dimensionless shape factor, λ is the x-ray wavelength, β is the line broadening at FWHM after subtracting the instrumental line broadening (in radians), θ is the Bragg angle, C depends on the assumptions made concerning the nature of ε, the inhomogeneous strain. We create Williamson-Hall plots to understand the components of peak broadening for the MOF samples of interest, where the slope is equal to the strain component, and the size component from the intercept (see Supplementary Fig. 4.6). Supplementary Table 4.1 shows the results from the Scherrer and Williamson-Hall analysis. We see that the MOFs grown on the Au substrates contain larger crystallite sizes compared to the MOFs grown on Al substrates, however the domain sizes are on the order of hundreds of nanometers which is orders of magnitude greater than the average MFP in the MOFs[34]. Thus, the increased grain boundaries do not explain their reduced thermal conductivity compared to the MOFs grown on Al substrates.

Supplementary Table 4.1 Results for the Scherrer and Williamson-Hall analysis on the XRD data of 300 nm HKUST-1 MOFs grown on Al and Au substrates.

	**HKUST-1/Al/SiO_2**	**HKUST-1/Au/SiO_2**
K	0.9	0.9
λ (Å)	1.5406	1.5406
Instrument Broadening (rad)	1.54e-4	1.54e-4
Average Crystallite Size (nm)	95.56	174.74
Strain ($C\varepsilon$)	0.00325	0.01458

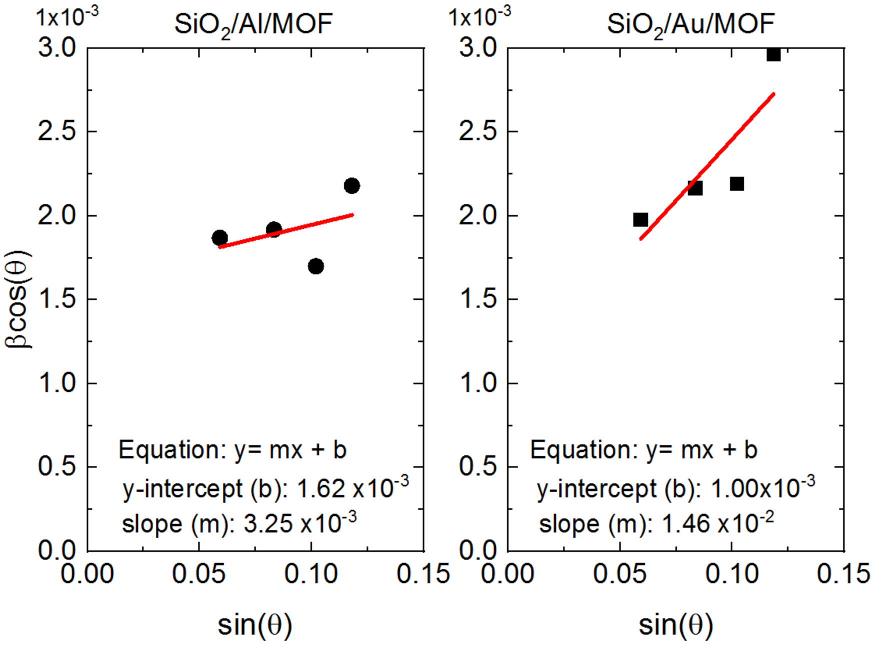

Supplementary Figure 4.6 Williamson-Hall plots derived from the XRD plots of 300 nm HKUST-1 SURMOFs deposited on Al/SiO_2 and Au/SiO_2 substrates respectively. The slope of the line is equal to the strain ($C\varepsilon$) in the samples, and the y-intercept is indicative of the domain size ($\frac{K\lambda}{L}$).

Supplementary Note 5. Frequency-domain thermoreflectance (FDTR) measurements

High-quality HKUST-1 single crystals were obtained from the Farha group at Northwestern University. The average crystal size is approximately 150 microns based on optical microscope images. Because it is important to have large, flat and smooth surfaces for laser-based frequency domain thermoreflectance (FDTR) measurements, good candidate crystals are carefully selected under the optical microscope. FDTR is a non-contact optical technique where continuous wave pump and probe lasers are used to measure thermal transport. The pump laser beam (488 nm) is intensity modulated by an electro-optic modulator at different frequencies and acts as a periodic heat source when it is absorbed at the sample surface. This periodic heating causes oscillations of the temperature on the surface at the same frequency as the pump beam, but with a phase lag due to the sample's inherent thermal impedance. The probe laser (532 nm) is co-aligned with the pump beam and measures the change in optical signal which is directly related to the temperature change of the sample based on thermoreflectance[23,35,36]. The temperature change of the sample is affected by the sample's thermal properties and is denoted in the phase-lag between the incoming pump and reflected probe lasers. The experimental phase-lag, as a function of pump modulation frequency, are fitted by an analytical solution to the three-dimensional dimensional heat diffusion equation for periodic surface heating of a layered solid by a radially Gaussian source[23]. The best-fit is achieved where the only fitting parameter is the desired sample thermal property – in this case, the HKUST-1 thermal conductivity.

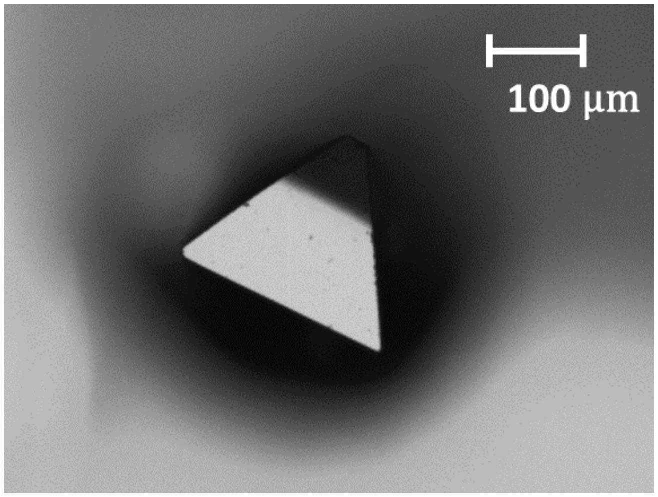

Supplementary Figure 5.1 10X optical microscope image of the HKUST-1 MOF sample partially covered with Au-Pt layer.

In FDTR measurements, optical lasers are converted to a thermal source by the transducer layer. Typically, a gold (Au) layer of ~60 nm thick is used for this purpose, but our sensitivity analysis indicated that we gain more sensitivity on the change in thermal conductivity of MOF crystals when we have a lower thermal conductivity transducer layer. Therefore, we lowered the thermal conductivity of the transducer by layering Au with platinum (Pt) due to Pt's low thermal conductivity and we specifically used a 30 nm Au (top) - 20 nm Pt (bottom) stacked transducer layer. The Au-Pt layer is sputtered on top of the HKUST-1 crystals at 1×10^{-5} Torr base pressure with an argon flow of 5×10^{-3} pressure and 25 sccm flow rate at room temperature. To ensure liquid adsorbates can enter the inner HKUST-1 crystal structure during adsorption measurements, a portion of the crystal is covered with a strip of Kapton tape under the optical microscope before sputtering. Supplementary Fig. 5.1 shows the optical microscope image of the HKUST-1 crystal that is partially covered with the Au-Pt layer.

S5.1 Fitting parameters

Supplementary Table 5.1 below shows fitting parameters that are used for fitting the experimental phase lags to the analytically calculated values. "Fitting" indicates that they are the unknown fitting parameters.

Supplementary Table 5.1 Material properties used for fitting in each case.

**Layer materials**	**Thermal conductivity (W/m·K)***	**Heat capacity (J/kg·K)**	**Density (g/cm^3)**	**Length (m)**
**Au (top)**	80 ± 15**	126 ± 3	19.3 ± 0.4	(30 ± 2) E -9
**Pt**	80 ± 15**	133 ± 2	21.4 ± 0.1	(20 ± 2) E -9
**Activated (crystal)**	0.69 ± 0.21*	775 ± 8***	0.88 ± 0.1	(150 ± 10) E -6
**Ethanol (crystal)**	0.29 ± 0.06*	1,430 ± 140***	1.43 ± 0.1	(150 ± 10) E -6
**Methanol (crystal)**	0.15 ± 0.02*	1,430 ± 140***	1.44 ± 0.1	(150 ± 10) E -6
**Water (crystal)**	0.21 ± 0.04*	2,290 ± 230***	1.58 ± 0.1	(150 ± 10) E -6

***Obtained through fitting. **Au and Pt thermal conductivities were determined from the Wiedemann-Franz law and electrical conductivity measurements of co-sputtered glass samples. ***Identical to the values in Supplementary Fig. 4.2**

Au and Pt thermal conductivities were determined from the Wiedemann-Franz law and electrical conductivity measurements of co-sputtered glass samples. For Au-Pt layer, the same thermal conductivity is assumed between Au and Pt as both are highly conductivity metals yet very thin. The actual thickness of the transducer layer is measured using X-ray reflectivity (XRR) technique and that of the crystal is assumed from the average crystal size from optical microscope images. The heat capacity of the activated HKUST-1 crystal before liquid adsorption was co-fit with the thermal conductivity and the combination that resulted in the lowest mean square error between data and experiment is reported. It is encouraging that this value of heat capacity agrees with that acquired by the thin film experiments in Supplementary Figs. 4.1-4.2, and with published experimental values[37,38]. Theoretically calculated values of heat capacity and density were used for each liquid adsorption case. We found that if the heat capacity and density were left at the activated (un-filled) values, the FDTR data could not be fit, which provides certainty that the filled values reported in Supplementary Table 5.1 are correct. Supplementary Fig. 5.2 shows that the analytical fit that assumes the theoretically calculated heat capacity for methanol filled HKUST-1 from Supplementary Table 5.1 (and Supplementary Fig 4.2) is far better than the fit found using the heat capacity of the activated unfilled HKUST-1. The uncertainties in heat capacity and density in HKUST crystal are set conservatively at 10% due to a lack of experimental data available for these properties.

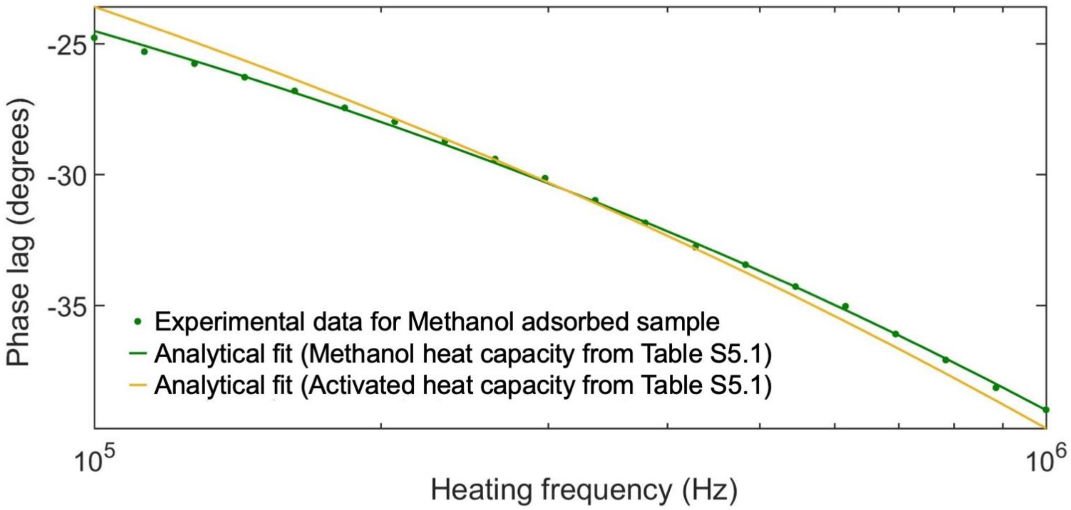

Supplementary Figure 5.2 FDTR Phase lag data vs. frequency for the methanol adsorbed sample.

S5.2. Thickness analysis

The X-ray reflectivity (XRR) fit agreed very well with the original data as presented in Supplementary Fig. 5.3. The fitted result indicated that the Pt thickness is 21 nm and Au thickness is 30 nm. When these thicknesses changed by 2 nm, the quality of fit worsened appreciably.

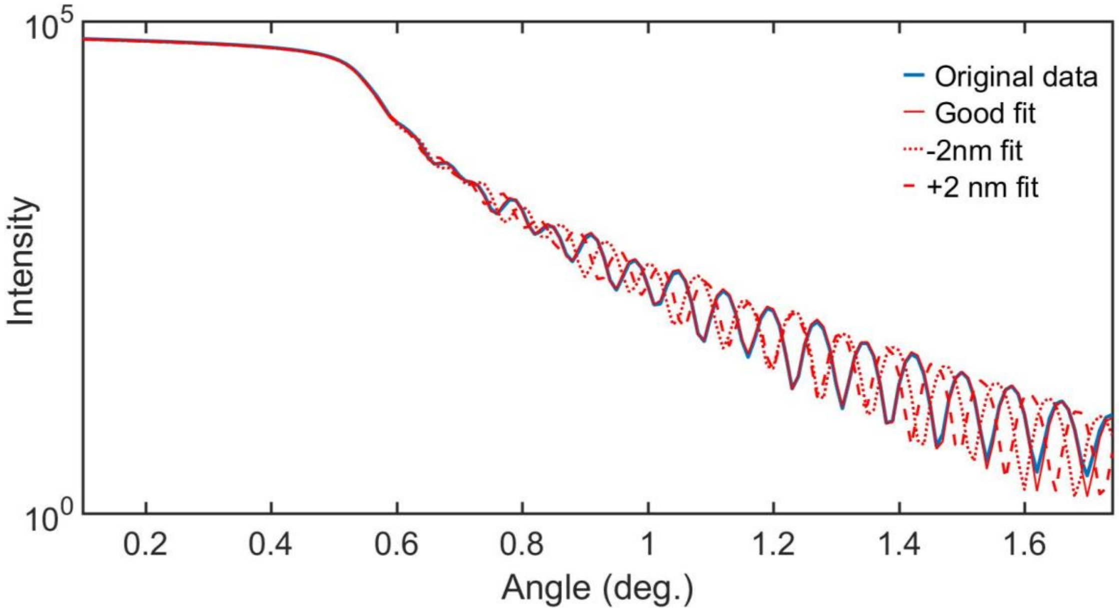

Supplementary Figure 5.3 The XRR thickness analysis result. The -2 nm and +2 nm lines show the sensitivity in fits with ± 2 nm change in the Au layer.

S5.3. Sensitivity analysis

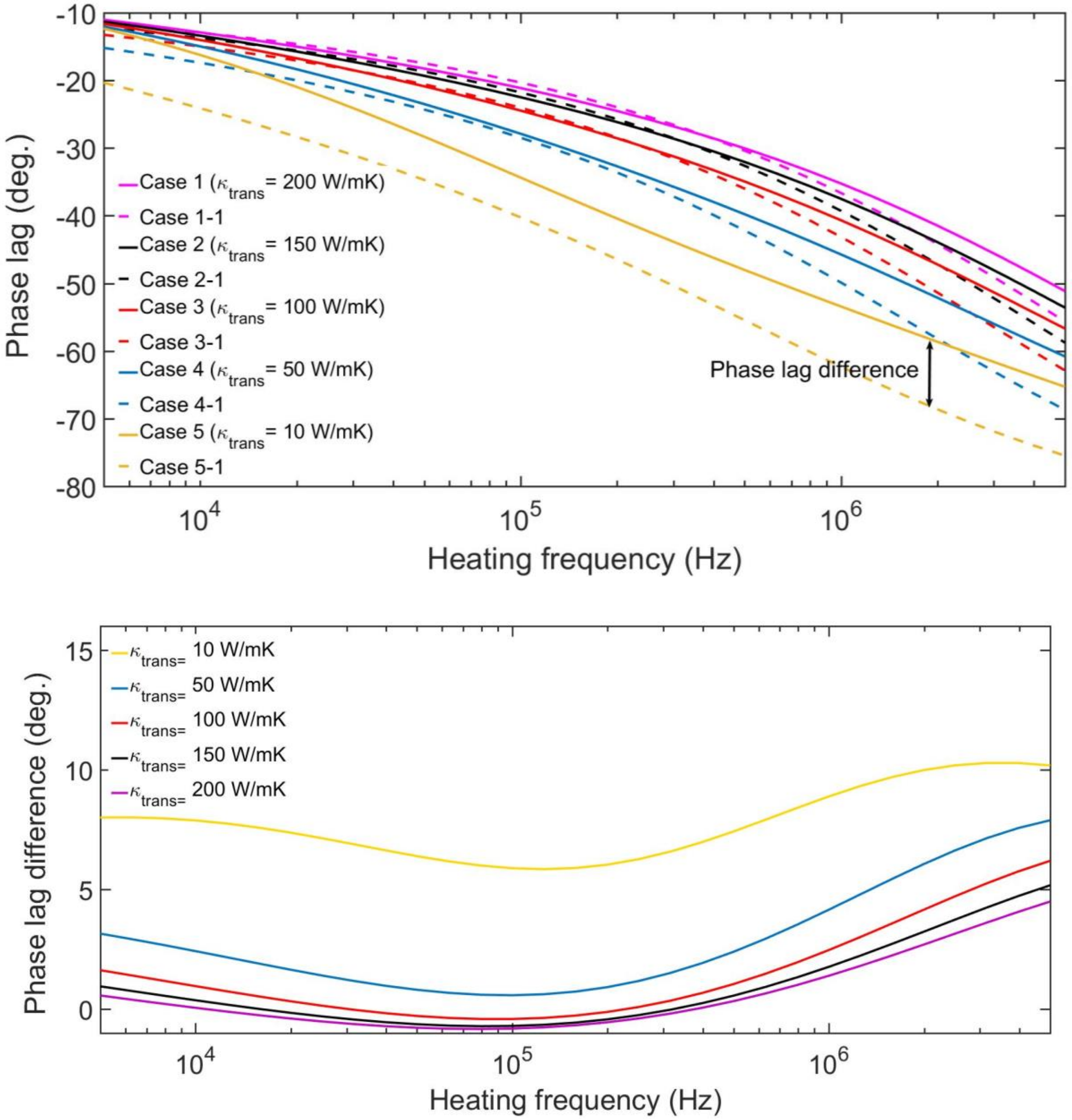

Supplementary Figure 5.4 (top) Analytically calculated phase lags as a function of heating frequency for different κ_{trans} when the MOF thermal conductivity changed from 0.7 W/m·K (solid lines) to 0.1 W/m·K (dashed lines). (bottom) The difference in phase lags represented with the solid and dashed lines as a function of heating frequency.

The phase lags as a function of heating frequency are analytically calculated when the thermal conductivity of MOF crystal is assumed to be 0.7 W/m·K and 0.1 W/m·K for different top transducer layer thermal conductivities (κ_{trans}). The calculation results are shown in Supplementary Fig. 5.4(top). The transducer layer thermal conductivity ranged between 10 W/m·K (Case 5) and 200 W/m·K (Case 1) where the solid lines represent the 0.7 W/m·K case and the dashed lines represent the 0.1 W/m·K case.

Supplementary Fig. 5.4(bottom) shows the difference in phase lags between the higher and lower MOF thermal conductivity cases for different κ_{trans} as a function of heating frequency. It is clear that the lower κ_{trans} yields a higher phase lag difference, which is equivalent to higher sensitivity for measuring the thermal properties of MOF crystals.

S5.4. Measurements data table

Data for the loaded single crystal HKUST-1 MOFs are listed in Supplementary Table 5.2. Different numbers in crystals and data points in the second and third columns mean that the data collection is performed on different crystals and multiple spots on a given crystal. The uncertainties in each data point in the last column resulted from the propagation of uncertainties in material properties used for fitting. Supplementary Fig. 5.5 shows a histogram of all the measurement results of the activated HKUST-1 crystal samples to show that the whole data sets did not yield severely scattered κ. This data set represents a total of 8 crystals and 21 measurement spots.

Supplementary Table 5.2 Selected measured κ of HKUST-1 crystals under different conditions.

Activated HKUST	Crystal 1	Data point 1	0.72 ± 0.27 W/m·K
		Data point 2	0.69 ± 0.26 W/m·K
		Data point 3	0.69 ± 0.27 W/m·K
	Crystal 2	Data point 1	0.75 ± 0.24 W/m·K
		Data point 2	0.61 ± 0.25 W/m·K
Methanol	Crystal 1	Data point 1	0.31 ± 0.24 W/m·K
		Data point 2	0.28 ± 0.26 W/m·K
		Data point 3	0.25 ± 0.23 W/m·K
		Data point 4	0.34 ± 0.33 W/m·K
Ethanol	Crystal 1	Data point 1	0.15 ± 0.08 W/m·K
		Data point 2	0.16 ± 0.08 W/m·K
Water	Crystal 1	Data point 1	0.19 ± 0.09 W/m·K
		Data point 2	0.24 ± 0.09 W/m·K

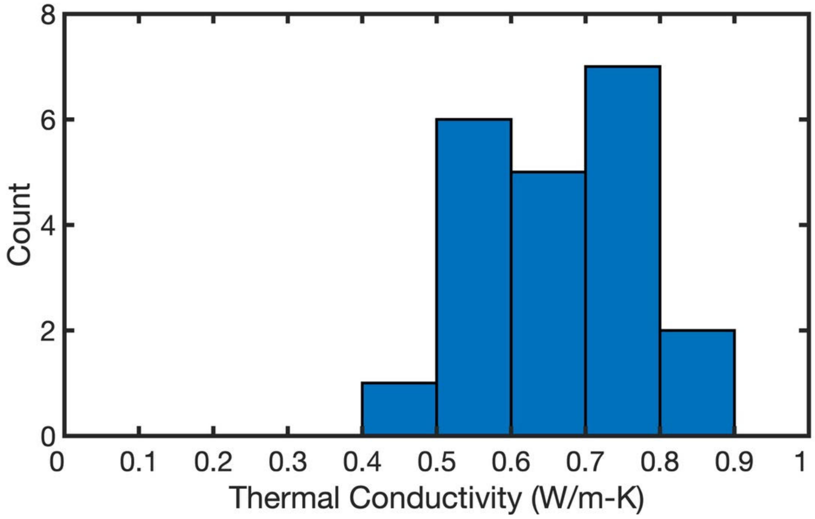

Supplementary Fig. 5.5 A histogram of thermal conductivity measurements of pristine HKUST-1 crystals after thermal activation. This data set represents a total of 8 crystals and 21 measurement spots.

Supplementary Note 6. Sintered sphere model for thermal conductivity

We derived an analytical model for thermal conductivity based on sintered spheres. This model examines the reduced thermal conductivity of the adsorbates within the infiltrated MOFs by considering the geometry of the liquids after MOF adsorption, where the adsorbed liquids can be thought of as point contacts where channels between the pores are similar to the necks between particles characteristic of sintered spheres made from compacted powders. In this model, we start with the total thermal conductivity (κ_T) of the guest@HKUST-1 system represented as the sum of two parallel resistors

$$\kappa_T = (1-\varphi)\kappa_{HKUST-1} + \kappa_{Liq_{eff}}, \quad \text{(S6.1)}$$

where $\kappa_{Liq_{eff}} = \varphi\kappa_{Liq}$ and again, φ is the void fraction occupied by the liquid. The porosity of the is 67.8% and we estimate the water void fraction to be 82% of the pores for the H_2O@HKUST-1, while the methanol and ethanol infiltrated are estimated at 100% void fraction[39]. This representation is accurate only if the liquid is oriented as slabs or rods that run parallel to the direction of heat flow, otherwise $\kappa_{Liq_{eff}} = 0$ (see Supplementary Fig. 6.1). Since the real geometry of the liquid is likely something in between these two cases, due to the many channels connecting the pores within the HKUST-1, we consider the case where the liquid occupies voids that are somewhat spherical (see Supplementary Fig. 6.1), contact one another, and apply models created for sintered spheres[40]. By treating the infiltrated liquid molecules as sintered spheres, we can understand how the geometry imposed by the MOF pores is reducing the thermal conductivity of the liquid. Specifically, we calculate the Reimann-Weber equation to derive the total effective contact conductivity (κ_c) of a simple cubic structure of equally overlapped spheres[41].

$$\kappa_c = 6\kappa_T\left(\frac{1}{x} + \frac{1}{\pi}\ln\left({}^{2}/_{x}\right)\right)^{-1}, \quad \text{(S6.2)}$$

where κ_T is defined above, and $x = a/R_s$ is the contact size ratio where a is the contact spot radius between two spheres and R_s is the sphere's radius. Supplementary Fig. 6.1 illustrates how these parameters were identified within the MOF system. We multiply by six because each modeled sphere contains six contact points. For actual spherical sintered spheres, the contact spot radius (a) is a function of the particles Young's modulus (E), Poisson ratio (ν), forces between the spheres (F), and the spot radius (R_s), shown in Eq. S6.3.

$$a = \sqrt[3]{\frac{6F\left[\frac{1-\nu_{MOF}^2}{E_{MOF}}\right]}{4\left(\frac{2}{R_S}\right)}} \qquad \text{(S6.3)}$$

Since it is difficult to define a for our system, we simplify our assumptions by solving Eq. S6.2 for a variety of contact size ratios (x), and set an upper and lower limit for x that would describe the reduction in thermal conductivity that is seen experimentally upon infiltration of the guest liquid molecules. The parameters used for this calculation are shown in Supplementary Table 6.1. We take the radius to be the radius of the largest pore in the MOF. Supplementary Fig. 6.2 shows a plot of the Reimann-Weber model for x vs. the normalized thermal conductivity (κ_c/κ_{MOF}). The shaded region in the figure indicates the range of x that would reduce the thermal conductivity of the guest@MOF system to the experimentally observed values assuming that the reduction in the total thermal conductivity is driven by the reduction to just the liquid's thermal conductivity. We see that if we use a value of R_s equal to the radius of the largest pore in the MOF, the range of contact spot radii that corresponds to the reduction in thermal conductivity are so small that they are unphysical. Therefore, we conclude that although the geometry of the MOF may be reducing the thermal conductivity of the liquid material, it is not the dominating mechanism for reducing the thermal conductivity of the guest@MOF system.

Supplementary Table 6.1: Parameters used for the Reimann-Weber sintered sphere calculations.

	Water@HKUST-1	Methanol@HKUST-1	Ethanol@HKUST-1
κ_T ($Wm^{-1}K^{-1}$)	0.567	0.363	0.341
R_s (nm)	0.575	0.575	0.575
a (nm)	0.03-0.08	0.04-0.13	0.04-0.14

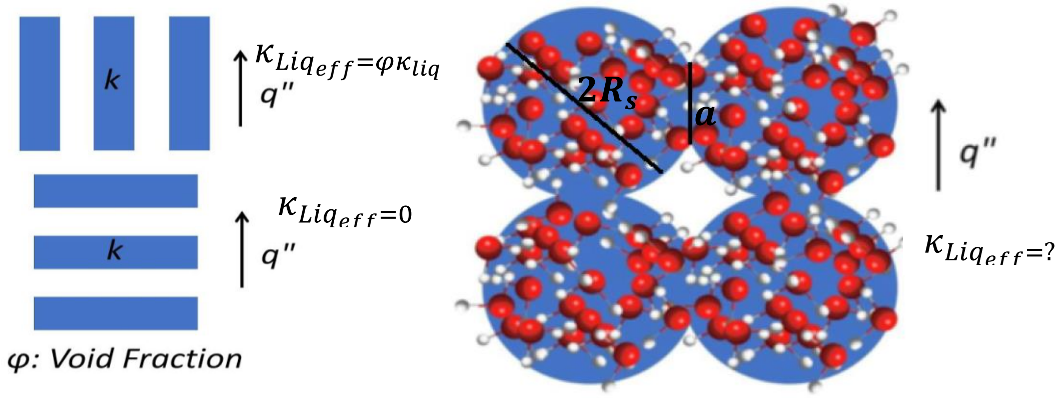

Supplementary Figure 6.1 Orientation of water compared to heat flow and its effect on $\kappa_{Liq_{eff}}$ (left). H2O@HKUST-1 SURMOF represented as sintered spheres (right).

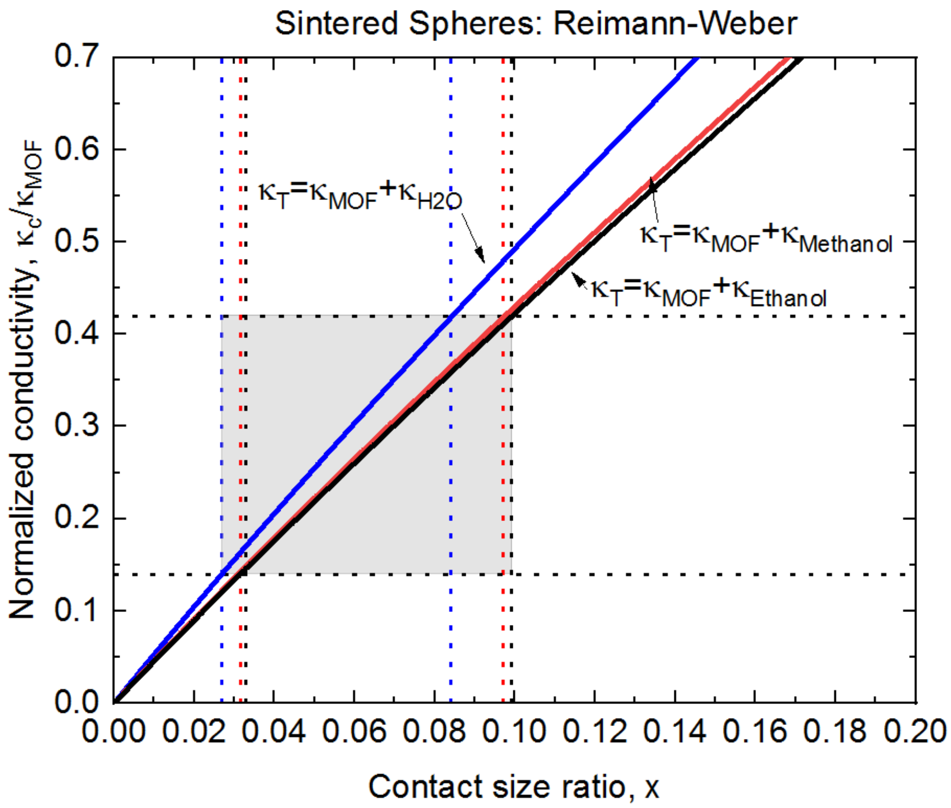

Supplementary Figure 6.2 Normalized thermal conductivity for the sintered sphere model. The shaded region shows the values of x required to reduce the thermal conductivity of the liquid to reach the thermal conductivity values observed experimentally ($y = \frac{\kappa_c}{\kappa_{MOF}} = 0.42 - 0.14$). The labelled lines correspond to the value of κ_T used for the Reimann-Weber calculations.

References

1. Chui, S. S.-Y., Lo, S. M.-F., Charmant, J. P. H., Orpen, A. G. & Williams, I. D. A Chemically Functionalizable Nanoporous Material $[Cu_3(TMA)_2(H_2O)_3]^n$. *Science* **283,** 1148–1150 (1999).
2. Zhao, L., Yang, Q., Ma, Q., Zhong, C., Mi, J. & Liu, D. A force field for dynamic Cu-BTC metal-organic framework. *J. Mol. Model.* **17,** 227–234 (2010).
3. Snurr, R. Q., Bell, A. T. & Theodorou, D. N. Prediction of adsorption of aromatic hydrocarbons in silicalite from grand canonical Monte Carlo simulations with biased insertions. *J. Phys. Chem.* **97,** 13742–13752 (1993).
4. Martin, M. G. & Siepmann, J. I. Transferable Potentials for Phase Equilibria. 1. United-Atom Description of n-Alkanes. *J. Phys. Chem. B* **102,** 2569–2577 (1998).
5. Chen, B., Potoff, J. J. & Siepmann, J. I. Monte Carlo Calculations for Alcohols and Their Mixtures with Alkanes. Transferable Potentials for Phase Equilibria. 5. United-Atom Description of Primary, Secondary, and Tertiary Alcohols. *J. Phys. Chem. B* **105,** 3093–3104 (2001).
6. Zhang, X. B., Liu, Q. L. & Zhu, A. M. An improved fully flexible fixed-point charges model for water from ambient to supercritical condition. *Fluid Phase Equilibria* **262,** 210–216 (2007).
7. Berendsen, H. J. C., Grigera, J. R. & Straatsma, T. P. The missing term in effective pair potentials. *J. Phys. Chem.* **91,** 6269–6271 (1987).
8. Darkrim, F. & Levesque, D. Monte Carlo simulations of hydrogen adsorption in single-walled carbon nanotubes. *J. Chem. Phys.* **109,** 4981–4984 (1998).
9. Allen, M. P. & Tildesley, D. J. *Computer Simulation of Liquids*. (Oxford University Press, 1989).
10. Plimpton, S. Fast Parallel Algorithms for Short-Range Molecular Dynamics. *J. Comput. Phys.* **117,** 1–19 (1995).
11. Babaei, H., Keblinski, P. & Khodadadi, J. M. Equilibrium molecular dynamics determination of thermal conductivity for multi-component systems. *J. Appl. Phys.* **112,** 054310 (2012).
12. Dubbeldam, D., Calero, S., Ellis, D. E. & Snurr, R. Q. RASPA: Molecular simulation software for adsorption and diffusion in flexible nanoporous materials. *Mol. Simul.* **42,** 81–101 (2016).
13. Gale, J. D. GULP: A computer program for the symmetry-adapted simulation of solids. *J. Chem. Soc. Faraday Trans.* **93,** 629–637 (1997).
14. Thomas, J. A., Turney, J. E., Iutzi, R. M., Amon, C. H. & McGaughey, A. J. H. Predicting phonon dispersion relations and lifetimes from the spectral energy density. *Phys. Rev. B* **81,** 081411 (2010).
15. Arslan, H. K., Shekhah, O., Wieland, D. C. F., Paulus, M., Sternemann, C., Schroer, M. A., Tiemeyer, S., Tolan, M., Fischer, R. A. & Wöll, C. Intercalation in Layered Metal–Organic Frameworks: Reversible Inclusion of an Extended π-System. *J. Am. Chem. Soc.* **133,** 8158–8161 (2011).
16. Gu, Z.-G., Pfriem, A., Hamsch, S., Breitwieser, H., Wohlgemuth, J., Heinke, L., Gliemann, H. & Wöll, C. Transparent films of metal-organic frameworks for optical applications. *Microporous Mesoporous Mater.* **211,** 82–87 (2015).
17. Gu, Z.-G., Heinke, L., Wöll, C., Neumann, T., Wenzel, W., Li, Q., Fink, K., Gordan, O. D. & Zahn, D. R. T. Experimental and theoretical investigations of the electronic band structure of metal-organic frameworks of HKUST-1 type. *Appl. Phys. Lett.* **107,** 183301 (2015).

18. Arslan, H. K., Shekhah, O., Wohlgemuth, J., Franzreb, M., Fischer, R. A. & Wöll, C. High-throughput fabrication of uniform and homogenous MOF coatings. *Adv. Funct. Mater.* **21,** 4228–4231 (2011).

19. Zhuang, J.-L., Terfort, A. & Wöll, C. Formation of oriented and patterned films of metal–organic frameworks by liquid phase epitaxy: A review. *Coord. Chem. Rev.* **307,** 391–424 (2016).

20. Müller, K., Fink, K., Schöttner, L., Koenig, M., Heinke, L. & Wöll, C. Defects as Color Centers: The Apparent Color of Metal–Organic Frameworks Containing Cu2+-Based Paddle-Wheel Units. *ACS Appl. Mater. Interfaces* **9,** 37463–37467 (2017).

21. Zhou, W., Wöll, C. & Heinke, L. Liquid- and Gas-Phase Diffusion of Ferrocene in Thin Films of Metal-Organic Frameworks. *Materials* **8,** 3767–3775 (2015).

22. Lide, D. R. *CRC Handbook of Chemistry and Physics, 85th Edition*. (CRC Press, 2004).

23. Cahill, D. G. Analysis of heat flow in layered structures for time-domain thermoreflectance. *Rev. Sci. Instrum.* **75,** 5119–5122 (2004).

24. Schmidt, A. J. Pump-probe thermoreflectance. *Annu. Rev. Heat Transf.* **16,** 159–181 (2013).

25. Hopkins, P. E., Kaehr, B., Phinney, L. M., Koehler, T. P., Grillet, A. M., Dunphy, D., Garcia, F. & Brinker, C. J. Measuring the Thermal Conductivity of Porous, Transparent SiO2 Films With Time Domain Thermoreflectance. *J. Heat Transf.* **133,** 061601-061601–8 (2011).

26. Schmidt, A. J., Chen, X. & Chen, G. Pulse accumulation, radial heat conduction, and anisotropic thermal conductivity in pump-probe transient thermoreflectance. *Rev. Sci. Instrum.* **79,** 114902–114902 (2008).

27. Hopkins, P. E. & Duda, J. C. Heat Transfer - Mathematical Modelling, Numerical Methods and Information Technology. 305–330 (2011).

28. Hopkins, P. E., Serrano, J. R., Phinney, L. M., Kearney, S. P., Grasser, T. W. & Harris, C. T. Criteria for cross-plane dominated thermal transport in multilayer thin film systems during modulated laser heating. *J. Heat Transf.* **132,** 081302–081302 (2010).

29. Thomsen, C., Strait, J., Vardeny, Z., Maris, H. J., Tauc, J. & Hauser, J. J. Coherent phonon generation and detection by picosecond light pulses. *Phys. Rev. Lett.* **53,** 989–992 (1984).

30. Giri, A., Niemelä, J.-P., Szwejkowski, C. J., Karppinen, M. & Hopkins, P. E. Reduction in thermal conductivity and tunable heat capacity of inorganic/organic hybrid superlattices. *Phys. Rev. B* **93,** 024201 (2016).

31. Meyer, K. E., Cheaito, R., Paisley, E., Shelton, C. T., Braun, J. L., Maria, J.-P., Ihlefeld, J. F. & Hopkins, P. E. Crystalline coherence length effects on the thermal conductivity of MgO thin films. *J. Mater. Sci.* **51,** 10408–10417 (2016).

32. Costescu, R. M., Wall, M. A. & Cahill, D. G. Thermal conductance of epitaxial interfaces. *Phys. Rev. B* **67,** 054302–054302 (2003).

33. Williamson, G. K. & Hall, W. H. X-ray line broadening from filed aluminium and wolfram. *Acta Metall.* **1,** 22–31 (1953).

34. Wang, X., Guo, R., Xu, D., Chung, J., Kaviany, M. & Huang, B. Anisotropic Lattice Thermal Conductivity and Suppressed Acoustic Phonons in MOF-74 from First Principles. *J. Phys. Chem. C* **119,** 26000–26008 (2015).

35. Regner, K. T., Sellan, D. P., Su, Z., Amon, C. H., McGaughey, A. J. H. & Malen, J. A. Broadband phonon mean free path contributions to thermal conductivity measured using frequency domain thermoreflectance. *Nat. Commun.* **4,** 1640 (2013).

36. Malen, J. A., Baheti, K., Tong, T., Zhao, Y., Hudgings, J. A. & Majumdar, A. Optical Measurement of Thermal Conductivity Using Fiber Aligned Frequency Domain Thermoreflectance. *J. Heat Transf.* **133,** 081601 (2011).

37. Ongari, D., Boyd, P. G., Barthel, S., Witman, M., Haranczyk, M. & Smit, B. Accurate Characterization of the Pore Volume in Microporous Crystalline Materials. *Langmuir* **33,** 14529–14538 (2017).

38. Mu, B. & Walton, K. S. Thermal Analysis and Heat Capacity Study of Metal–Organic Frameworks. *J. Phys. Chem. C* **115,** 22748–22754 (2011).

39. Van Assche, T. R. C., Duerinck, T., Gutiérrez Sevillano, J. J., Calero, S., Baron, G. V. & Denayer, J. F. M. High Adsorption Capacities and Two-Step Adsorption of Polar Adsorbates on Copper–Benzene-1,3,5-tricarboxylate Metal–Organic Framework. *J. Phys. Chem. C* **117,** 18100–18111 (2013).

40. Gusarov, A. V., Laoui, T., Froyen, L. & Titov, V. I. Contact thermal conductivity of a powder bed in selective laser sintering. *Int. J. Heat Mass Transf.* **46,** 1103–1109 (2003).

41. Luikov, A. V. *Heat and Mass Transfer Energia Handbook*. (1971).
